# Analysis of three genomes within the thermophilic bacterial species *Caldanaerobacter subterraneus* with a focus on carbon monoxide dehydrogenase evolution and hydrolase diversity

**DOI:** 10.1186/s12864-015-1955-9

**Published:** 2015-10-07

**Authors:** FH Sant’Anna, AV Lebedinsky, TG Sokolova, FT Robb, JM Gonzalez

**Affiliations:** Institute of Natural Resources and Agrobiology, Spanish Council for Research, IRNAS-CSIC, Avda. Reina Mercedes 10, 41012 Sevilla, Spain; CAPES Foundation, Ministry of Education of Brazil, Brasília, DF 70040-020 Brazil; Winogradsky Institute of Microbiology, Russian Academy of Sciences, Prospect 60-letiya Oktyabrya 7/2, 117312 Moscow, Russia; Department of Microbiology and Immunology, University of Maryland and Institute of Marine and Environmental Technology, 701 E Pratt Street, Baltimore, MD 21202 USA

**Keywords:** Caldanaerobacter subterraneus, Genome, Horizontal gene transfer, Hydrogenase, Carbon monoxide dehydrogenase, Glycosidase, Protease, Esterase, Phylogeny, Thermophile

## Abstract

**Background:**

The *Caldanaerobacter subterraneus* species includes thermophilic fermentative bacteria able to grow on carbohydrates substrates with acetate and L-alanine as the main products. In this study, comprehensive analysis of three genomes of *C. subterraneus s*ubspecies was carried in order to identify genes encoding key metabolic enzymes and to document the genomic basis for the evolution of these organisms.

**Methods:**

Average nucleotide identity and *in silico* DNA relatedness were estimated for the studied *C. subterraneus* genomes. Genome synteny was evaluated using R2CAT software. Protein conservation was analyzed using mGenome Subtractor. Horizontal gene transfer was predicted through the GOHTAM pipeline (using tetranucleotide composition) and phylogenetic analyses (by maximum likelihood). Hydrolases were identified through the MEROPS and CAZy platforms.

**Results:**

The three genomes of *C. subterraneus* showed high similarity, although there are substantial differences in their gene composition and organization. Each subspecies possesses a gene cluster encoding a carbon monoxide dehydrogenase (CODH) and an energy converting hydrogenase (ECH). The CODH gene is associated with an operon that resembles the *Escherichia coli* hydrogenase *hyc*/*hyf* operons, a novel genetic context distinct from that found in archetypical hydrogenogenic carboxydotrophs. Apart from the CODH-associated hydrogenase, these bacteria also contain other hydrogenases, encoded by *ech* and *hyd* genes. An Mbx ferredoxin:NADP oxidoreductase homolog similar to that originally described in the archaeon *Pyrococcus furiosus* was uniquely encoded in the *C. subterraneus* subsp. *yonseiensis* genome. Compositional analysis demonstrated that some genes of the CODH-ECH and *mbx* operons present distinct sequence patterns in relation to the majority of the other genes of each genome. Phylogenetic reconstructions of the genes from these operons and those from the *ech* operon are incongruent to the species tree. Notably, the *cooS* gene of *C. subterraneus* subsp. *pacificus* and its homologs in *C. subterraneus* subsp. *tengcongensis* and *C. subterraneus* subsp. *yonseiensis* form distinct clades. The strains have diverse hydrolytic enzymes and they appear to be proteolytic and glycolytic. Divergent glycosidases from 14 families, among them amylases, chitinases, alpha-glucosidases, beta-glucosidases, and cellulases, were identified. Each of the three genomes also contains around 100 proteases from 50 subfamilies, as well about ten different esterases.

**Conclusions:**

Genomic information suggests that multiple horizontal gene transfers conferred the adaptation of *C. subterraneus* subspecies to extreme niches throughout the carbon monoxide utilization and hydrogen production. The variety of hydrolases found in their genomes indicate the versatility of the species in obtaining energy and carbon from diverse substrates, therefore these organisms constitute a remarkable resource of enzymes with biotechnological potential.

**Electronic supplementary material:**

The online version of this article (doi:10.1186/s12864-015-1955-9) contains supplementary material, which is available to authorized users.

## Background

Thermophilic bacteria possess diverse adaptations in order to thrive under high temperatures [1, 2]. Therefore, these organisms are sources of potentially useful thermostable proteins, which is promising because of the increasing biotechnological interest in highly thermostable enzymes [3]. Besides, the genomic study of these organisms can provide insights on interesting metabolic features characteristic of these bacteria, like the ability to generate hydrogen gas as metabolic product, a promising renewable fuel. With the advent of high throughput technologies of DNA sequencing, many genomes of thermophilic bacteria are being unraveled (e.g. [4–7], and the in silico analysis of the large amount of generated data is a fundamental initial approach to understand the full potential of these organisms.

*Caldanaerobacter subterraneus* includes fermentative thermophilic bacteria with relatively low genomic GC content (under 40 %) able to grow on carbohydrate substrates with acetate L-alanine, H_2_, and CO_2_ as the main products that have been isolated from a variety of hot environments [8–11]. *C. subterraneus* subsp. *pacificus* (formerly known as *Carboxydobrachium pacificum*) is known to grow on CO hydrogenogenically [8]; *C. subterraneus* subsp. *tengcongensis*–formerly *Thermoanaerobacter tengcongensis*–and *C. subterraneus* subsp. *yonseiensis* (but not *C. subterraneus* subsp. s*ubterraneus*) have been reported to oxidize CO [11]; however there is no mention if they produce hydrogen from CO. In 2002, the genome of *C. subterraneus* subsp. *tengcongensis* was sequenced. A CODH gene *cooS* was found in the genome and ascribed to the acetogenic Wood-Ljungdahl pathway [12]. However, after this report it was noted that the genome lacks the acetyl-CoA synthase gene, indispensable for this pathway, and the CODH gene is clustered with ECH genes, suggesting that *C. subteraneus* subsp. *tencongensis* has the capacity for hydrogenogenic carboxydotrophy [13]. Recently, the genome of *C. subterraneus* subsp. *yonseiensis* has also been published [14], which can contribute to the understanding of the evolution of the metabolic features in this species relative to its sibling strains. Moreover, these genomes constitute helpful resources for cloning and expression of novel enzymes of biotechnological importance (e.g. [1, 2, 15]).

In this study, the genome of *C. subterraneus* subsp. *pacificus* was sequenced. This bacterium grows from 50 to 80 °C, and was isolated from a submarine thermal vent in Japan, unlike the other subspecies (*C. subterraneus* subsp. *tengcongensis* and *C. subterraneus* subsp. *yonseiensis* were isolated from terrestrial high temperature environments, and *C. subterraneus* subsp. *subterraneus* strains are oilfield isolates). *C. subterraneus* subsp. *pacificus* is known to be able to grow chemolithotrophically on CO, producing H_2_ and CO_2_ during growth [8].

The main objective of this study was to explore the differences among the three genomes by comparative analysis. The analyses were focused on inferring the physiological and evolutionary aspects of these organisms. The role of horizontal gene transfer (HGT) in shaping these three genomes was also evaluated and key metabolic genes and proteins with potential biotechnological application, such as carbon monoxide dehydrogenase, hydrogenases, proteases, glycosidases and esterases were identified.

## Results and discussion

### Phylogeny of the species

**Fig. 1:**
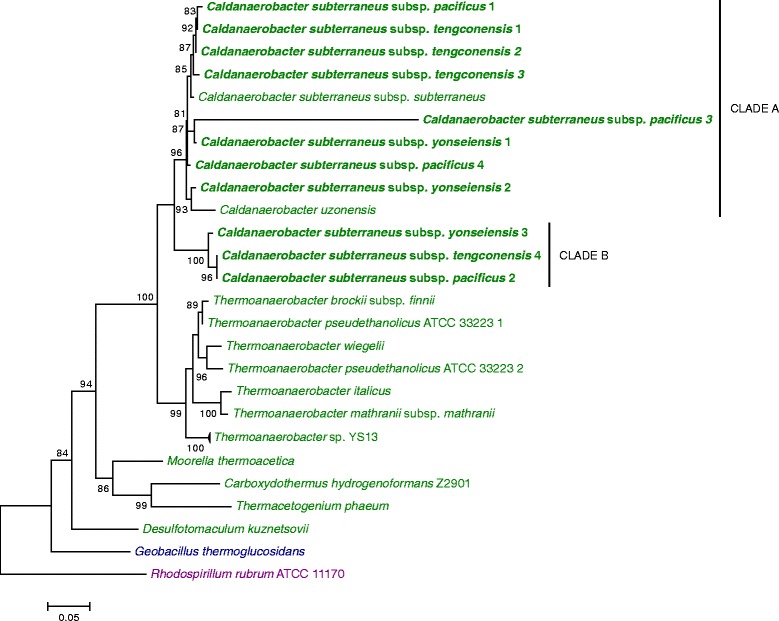
Evolutive history of *Caldanaerobacter subterraneu*s subspecies. The 16S rRNA tree was constructed using the maximum-likelihood method. aLRT values greater than 70 % are shown next to the branches. The tree is drawn to scale, with branch lengths in the same units as those of the evolutionary distances used to infer the phylogenetic tree. *Caldanaerobacter subterraneus* subspecies are in *bold*. Other Clostridiales species are in *green*, Bacillales species in *blue. R. rubrum* was used as outgroup (in *purple*)

A phylogenetic tree was constructed using 16S rRNA gene sequences. Thermoanaerobacterales and other bacterial species were included to demonstrate the evolutionary context of *C. subterraneus* subspecies, and to use them as reference for comparative purposes against other gene dendrograms. The tree included available copies of 16S rRNA genes of *Caldanaerobacter* species and subspecies (Fig. 1). The resulting 16S rRNA tree is in agreement with previous information: Sokolova et al. [8] and Subbotina et al. [16] have also shown that the species later reassigned to the genus *Caldanaerobacter* [11] are very close to each other and form a clade adjacent to but distinct from the clade of *Thermoanaerobacter* species.

*C. subterraneus* subsp. *tengcongensis* is known to exhibit an exceptionally high level of sequence divergence among its intragenomic 16S rRNA gene copies (6.7 %) [17]. As demonstrated in Fig. 1, the *C. subterraneus* subspecies with available genomes exhibit multiple 16S rRNA gene copies that are separated in two main clades (Clade A and Clade B, Fig. 1). This clade separation most probably represents the most ancient gene duplication that occurred before the diversification of subspecies. Considering Clade B, *C. subterraneus* subsp. *pacificus* is closer to *C. subterraneus* subsp. *tengcongensis* than to *C. subterraneus* subsp. *yonseiensis*. This pattern is not evident in Clade A due to the presence of intra-subspecies multiple 16S rRNA genes that interfere with interpretation of the true phylogenetic relationships among these subspecies.

### Genomes overview and horizontal gene transfer detection

As expected, the average nucleotide identity (ANI) and in silico prediction of in vitro DNA-DNA hybridization (DDH) values for the genomes of *C. subterraneus* subspecies confirmed the conclusion of Fardeau et al. [11] about the affiliation of the *C. subterraneus* subspecies within the same species and once more showed the closest proximity of *C. subterraneus* subsp. *pacificus* to *C. subterraneus* subsp. *tengcongensis* (ANI value was 98.8 % and DDH value 85 %) than to *C. subterraneus* subsp. *yonseiensis* (ANI value was 98.0 % and DDH value was 80 %).

**Fig. 2:**
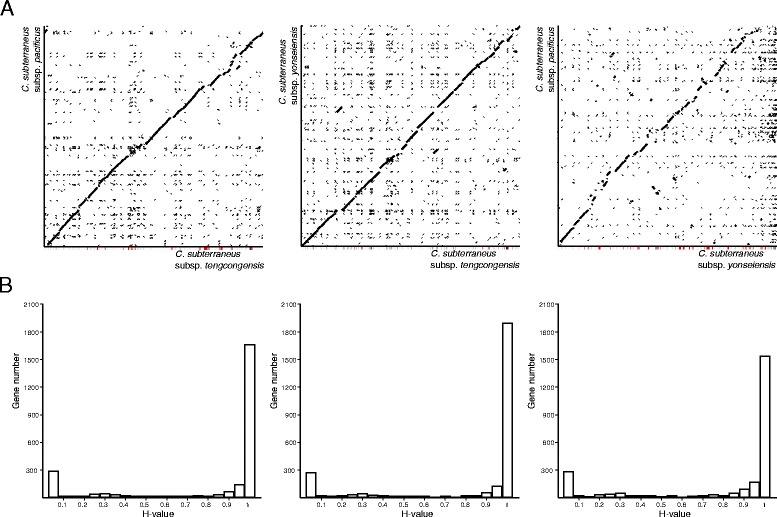
Comparison of *C. subterraneus* genomes. **a** Synteny plots demonstrating the high collinearity of the *C. subterraneus* genomes. *C. subterraneus* subsp. *pacificus* vs. *C. subterraneus* subsp. *tengcongensis* (*left*), *C. subterraneus* subsp. *yonseiensis* vs. *C. subterraneus* subsp. *tengcongensis* (*middle*), and *C. subterraneus* subsp. *pacificus* vs. *C. subterraneus* subsp. *yonseiensis* (*right*). **b** Histograms of H-values (a homology measure, see Methods) for all predicted proteins of *C. subterraneus* subspecies. *C. subterraneus subsp. pacificus* vs. *C. subterraneus subsp. tengcongensis* (*left*), *C. subterraneus subsp. yonseiensis* vs. *C. subterraneus subsp. tengcongensis* (*middle*), *and C. subterraneus subsp. pacificus* vs. *C. subterraneus subsp. yonseiensis* (*right*)

The genomes of *C. subterraneus* subsp. *pacificus* and *C. subterraneus* subsp. *yonseiensis* present a similar pattern of high colinearity with the *C. subterraneus* subsp. *tengcongensis* genome (Fig. 2a). These data are reinforced by the results of homology score (H-value) distribution of the CDSs, which shows a high number of common CDSs between *C. subterraneus* subsp. *tengcongensis* and the other two subspecies (Fig. 2b).

Table 1 shows a comparison of the general features of the three *C. subterraneus* genomes. Although these genomes all present low overall GC content (~37.7 %), their rRNAs and tRNAs have higher GC content (higher than 59.0 %), which corroborates the recognized positive correlation between the GC content of the rRNA and tRNA and optimal growth temperatures of prokaryotes [17].

**Table 1:** Overview of *C. subterraneus* genomes

	*C. subterraneus* subsp. *pacificus*	*C. subterraneus* subsp. *tengcongensis*	*C. subterraneus* subsp. *yonseiensis*
Genome size (Mb)	2.39	2.69	2.7
Genome GC content (%)	37.7	37.8	37.7
Number of contigs	135	1 (complete chromosome)	102
CDS	2511	2588	2711
Operon	871	1291	880
Hypothetical proteins^a^	962 (38.31 %)	855 (33.04 %)	836 (30.84 %)
Average gene length	819	905	834
rRNA	11	12	18
rRNA average GC content (%)	59.8	59.81	59.3
tRNA	49	56	59
tRNA average GC content (%)	60.26	60.12	59.98
Number of horizontally transferred CDSs^b^	173 (6.88 %)	121 (4.67 %)	127 (4.68 %)
Origin	Pacific Ocean hot vents	Terrestrial hot spring	Geothermal hot stream
Reference	This study	[12]	[14]

^a^Percentage of hypothetical proteins of all genome proteins is in parentheses

^b^Detected by GOHTAM. In parentheses is the percentage of horizontally transferred CDSs of all CDSs present in the genome

Genes that putatively could have been acquired via horizontal transfer were identified in all three genomes. In *C. subterraneus* subsp. *pacificus* and *C. subterraneus* subsp. *yonseiensis*, most of the putative horizontally transferred genes correspond to hypothetical proteins, 99 of 173 (57.2 %), and 75 of 127 (59.1 %), respectively. *C. subterraneus* subsp. *tengcongensis* presents a lower proportion of hypothetical genes that could have been horizontally transferred, 55 of 121 CDSs (45.5 %). Also, some of the xenologous CDSs are transposases (9 in *C. subterraneus* subsp. *tengcongensis*, 8 in *C. subterraneus* subsp. *pacificus*, and 5 in *C. subterraneus* subsp. *yonseiensis*).

### CODH dehydrogenase and Hyf/Hyc hydrogenase

Carbon monoxide dehydrogenases (CODH) are enzymes that catalyze the interconversion of CO and CO2, and they vary in their functional roles in the cell [18, 19]. All three *C. subterraneus* subspecies examined in this study possess a *cooS* gene encoding a CODH that is upstream of a hydrogenase gene cluster, with an invariant gene order identical to that found in *Geobacillus thermoglucosidans* strains [20].

**Fig. 3:**
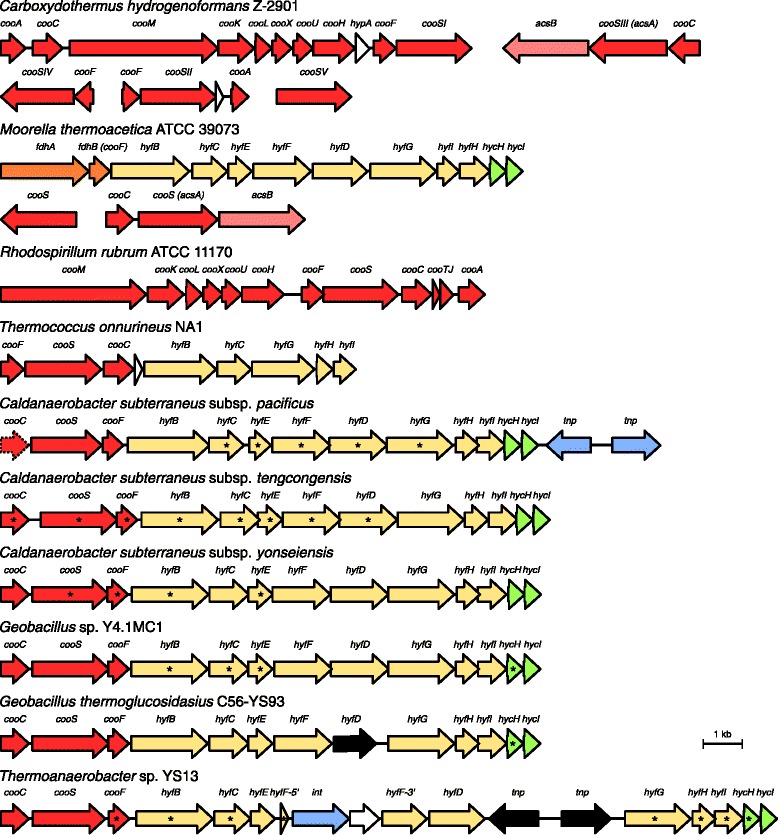
Comparison of the CODH-hydrogenase gene cluster organization between *C. subterraneus* and other species. *Arrows* represent genes and their respective direction of transcription. *Asterisks* indicate significant prediction of HGT in *C. subterraneus* and *Geobacillus thermoglucosidans* strains

In Fig. 3, the *cooS* genetic contexts of *C. subterraneus* subspecies and *G. thermoglucosidans* strains are contrasted to those from model organisms for studying carboxydotrophy, such as *Carboxydothermus hydrogenoformans*, *Moorella thermoacetica*, *Rhodospirillum rubrum* (Bacteria), and *Thermococcus onnurineus* (Archaea). The species *C. hydrogenoformans*, for example, possesses five *cooS* paralogs distributed along the genome, and their genetic contexts provide clues on the physiological roles of the CODHs in this organism [19]. As in *C. subterraneus*, in *R. rubrum* and *C. hydrogenoformans*, hydrogenase genes are also clustered with a *cooS* gene, and are identified by the prefix *coo*. In these organisms, it was suggested that the CODH and the *coo* hydrogenase gene cluster includes genes encoding proteins required for proton translocation, fundamental for energy conservation [21, 22]. Although the hydrogenase genes of *C. subterraneus* have homologous counterparts in the *R. rubrum* and *C. hydrogenoformans coo* hydrogenase genes, the former ones are more similar to the *hyf*/*hyc* genes from *Escherichia coli*, encoding the hydrogenase module of formate hydrogen lyase complexes [23]. A homologous *hyf*/*hyc* operon with identical genetic organization to that from *C. subterraneus* subspecies is also present in *M. thermoacetica* (Fig. 3), where it also includes formate dehydrogenase genes and it is thought to encode a formate hydrogen lyase complex [4]. In the archaeon *T. onnurineus*, the *cooS* gene is associated to *hyf*-*hyc* homologs (Fig. 3), which are fundamental for carboxydotrophic hydrogenogenesis [24, 25]. Interestingly, the organization of these hydrogenase genes is identical to that found in the *hyc* operon of *E. coli* [26] where the *hyfDEF* homologs are absent (in Fig. 3, the hydrogenase genes were named as *hyf* in order to permit a clear identification of the homologous genes among the considered species). The *hyc* and *hyf* operons encode for paralogous energy-converting Ni-Fe hydrogenases Hyd-3 and Hyd-4 of *E. coli*, which have significant similarity to the components of NADH:quinone oxidoreductase (complex I), suggesting their implication in energy metabolism [23]. Therefore, although distinct from the *coo* hydrogenase, it is likely that in *C. subterraneus* the Hyc/Hyf proteins and CODH form a complex responsible to extract energy by CO oxidation. This metabolism is often stated to be ancient, R. Hedderich [27], for example, suggested that energy-converting hydrogenases may have been originally associated with CODH.

Although Bao et al. [12] suggested that the CODH could also be utilized to fix carbon throughout the Wood-Ljungdahl pathway in *C. subterraneus* subsp. *tengcongensis*, it is improbable, because the genome of this strain does not present a gene putatively encoding for the key enzyme acetyl coenzyme A synthase (*acsB*) [4]. As well, this gene was not found in the other two subspecies of *C. subterraneus* suggesting that the same argument can be applied to all these three subspecies, which indicates a limitation for the use of carbon monoxide or carbon dioxide as a carbon source. This contrasts to the capabilities of other thermophilic, CO-utilizing, hydrogenogenic or acetogenic Firmicutes (e.g., *C. hydrogenoformans* [28] and *M. thermoacetica* [4]) (Fig. 3).

**Fig. 4:**
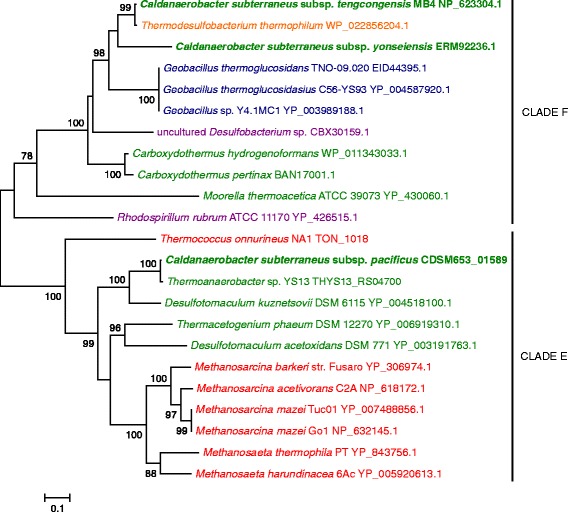
Evolutive history of CODH (CooS) from *C. subterraneus* subspecies. Details are as shown in Fig. 1, unless specified otherwise. *Thermodesulfobacterium* is in *orange*, and Archaea are in *red*. Accession number or locus tag are adjacent the species name. This tree is mid-point rooted. Classification of clades as in Techtmann et al. [29]

As demonstrated in Additional file 1: Figure S1, the CODHs of all three *C. subterraneus* subspecies contain the conserved amino acid residues important for the activity of this enzyme when compared to the archetypical CODHs deposited in PDB database. However, the CODH of *C. subterraneus* subsp. *pacificus* has important distinctive characteristics in relation to those of the other two subspecies of *C. subterraneus*, as the absence of the regions 450–454 and 537–544 (Additional file 1: Figure S1). In fact, this CODH has 66 % identity with its counterpart from *Methanosarcina acetivorans* (NP_618172.1), while in relation to those from *C. subterraneus* subsp. *tengcongensis* and *C. subterraneus* subsp. *yonseiensis* has 50 and 49 % identity, respectively. This observation, Blast searches and their alignments indicate that these proteins are not true orthologs but rather pseudoorthologs (xenologs). To investigate this finding, the phylogeny of these CODHs was analyzed. The resulting phylogenetic tree suggests a recent inter-phylum transfer of CODH gene from *C. subterraneus* subsp. *tengcongensis* to *Thermodesulfobacterium thermophilum* and possible more ancient CODH gene transference between Bacteria and Archaea (Fig. 4). The tree also confirmed that the CODHs of *Caldanaerobacter* subspecies can be classified in different lineages (Fig. 4).

Considering the species 16S rRNA tree as reference (Fig. 1), this result was unexpected. The CODH of *C. subterraneus* subsp. *pacificus* clusters with the homologous counterpart of *Thermoanaerobacter* sp. YS13 (having 98.8 % identity with this protein), and both are relatively distant from the CODHs from the other *C. subterraneus* subspecies. Following the classification described in Techtmann et al. [29], the CODH of *C. subterraneus* subsp. *pacificus* belongs to “Clade E”. On the other hand, the CODHs from *C. subterraneus* subsp. *tengcongensis* and *C. subterraneus* subsp. *yonseiensis* are immersed in “Clade F” (Fig. 4) and are rather closely related to the homologous proteins from *Geobacillus thermoglucosidans* strains (Order Bacillales), which are relatively distant from *Caldanaerobacter* (Order Clostridiales) (Fig. 1). However, despite their affiliation with the same clade, the CODHs of *C. subterraneus* subsp. *tengcongensis* and *C. subterraneus* subsp. *yonseiensis* are distant enough from each other to exclude their vertical inheritance from the LCA (last common ancestor) of these subspecies. While most of *C. subterraneus* subsp. *yonseiensis* proteins have H-values higher than 0.95 in relation to the *C. subterraneus* subsp. *tengcongensis* proteins (Fig. 2 and Additional file 2: Table S1), the CODH (O163_06470) has a H-value of 0.78 (Additional file 2: Table S1). These results suggest that the CODH evolutionary history of *C. subterraneus* includes several recent HGT events.

Taking these observations into account, all genes of the CODH-hydrogenase gene cluster were investigated for HGT by means of detection of phylogenetic discrepancies and parametric methods. Becq et al. [30] showed tetranucleotide composition and codon usage analyses had mean specificity of 87.8 and 89.2 %, and mean sensitivity of 77.2 and 91.5 %, respectively, when these methods were tested with artificial genomes. These values vary depending on intrinsic genome characteristics of the recipient organism, and on the origin of the HGT (e.g. a DNA sequence from a phylogenetically close related donor can be poorly detected by these methods). The greatest advantage of parametric methods is they do not rely on sequence data banks as phylogenetic approaches do [31]. However, phylogenetic reconstruction is necessary to infer historical events from sequences [32].

Parametric analysis of nucleotide composition revealed that some CODH-hydrogenase genes from *G. thermoglucosidans* and prominently those from *C. subterraneus* subspecies have differential sequence patterns in relation to the “standard” gene sequence pattern of each genome (Fig. 3 and Additional file 2: Table S1), which suggests that these genes could have been acquired by horizontal transfer. The presence of a transposase gene downstream the CODH-hydrogenase gene cluster in *C. subterraneus* subsp. *pacificus* represents an additional evidence supporting this hypothesis (Fig. 3).

The close relationship of the *hyf*/*hyc* hydrogenase gene cluster between *C. subterraneus* and *G. thermoglucosidans* strains was confirmed by phylogenetic analyses. Apparently, these *hyc* and *hyf* genes shared common evolutionary histories and were acquired together as a cluster, not individually (Fig. 3 and Additional file 2: Table S1). Considering the phylogenetic distance between *Geobacillus* and *C. subterraneus*, the high identity levels between their CODH-hydrogenase proteins (~70–80 %) can hardly be interpreted as a result of vertical inheritance from the LCA but are rather a result of acquisition of the cluster by the *C. subterraneus* lineage via HGT or its acquisition by both *C. subterraneus* and *G. thermoglucosidans* lineages from the same source or sources related to each other.

In general, in most of the phylogenetic trees of the CODH-hydrogenase gene cluster, the three subspecies of *C. subterraneus* form monophyletic clades with *Thermoanaerobacter* sp. YS13 (Additional file 3: Figure S2 and Additional file 4: Figure S3), with the exception of *cooF* and *coo*S genes (Fig. 4). In fact, the *hyc* and *hyf* genes of *Thermoanaerobacter* sp. YS13 have identity values higher than 96 % at the nucleotide level when compared to the *C. subterraneus* subsp. *tengcongensis* orthologous genes. These observations and the presence of genes implicated with transposition within its CODH-hydrogenase gene cluster (Fig. 4) support the idea that this strain inherited the CODH-hydrogenase gene cluster from *C. subterraneus*. Since *C. subterraneus* subsp. *pacificus* is closer to *C. subterraneus* subsp. *tengcongensis* than any other organism in most phylogenetic reconstructions (Fig. 1 and Additional file 4: Figure S3) and gene comparisons (*hyfCDEGHI* and *hycH* are identical at nucleotide sequence level), it is likely that the gene transfer event occurred before the diversification of these subspecies. This observation implies that most probably their LCA harbored a “Clade E” CODH (as *C. subterraneus* subsp. *pacificus*). In spite of our contention that the *hyf*/*hy*c hydrogenase gene cluster was acquired as a cluster, not as individual genes, there is evidence that the *cooF* and *co*o*S genes* from *C. subterraneus* have distinct evolutionary histories with respect to the other genes of the cluster. An important point is that the GC contents of *cooF* and *coo*S genes from *C. subterraneu*s subsp. *tengcongensis* (ca. 57 and 61 % respectively) and *C. subterraneu*s subsp. *yonseiensis* (ca. 51 and 49 % respectively) are much higher than the mean gene GC content of these genomes (ca. 38 %) (Additional file 2: Table S1). Together with the above-mentioned considerable differences in the amino acid and nucleotide sequence patterns, the GC content data suggests that the CODHs of *C. subterraneu*s subsp. *tengcongensis* and *C. subterraneu*s subsp. *yonseiensi*s were acquired recently via independent HGT events from prokaryotes having a higher genomic GC content after the diversification of the subspecies.

An important point to consider regarding the CODH tree is that its composition is very diverse taxonomically and does not reflect properly species relationships, indicating that HGT played an important role in the current distribution of carboxydotrophy among prokaryotes. Independent studies already pointed out that HGT of the *cooS* gene likely took place in several thermophilic species [29, 33, 34]. Despite the fact that the donor and acceptor organisms in these instances may be phylogenetically remote, they are usually able to grow in anaerobic environments at similar ranges of temperature and pH [33, 35]. The acquisition of new physiological characteristics would putatively allow the recipient organisms to be recruited to new thermophilic consortia, and consequently, the horizontal transference of important genes for adaptation to specialized niches would be facilitated.

Our analysis revealed at least four recent independent HGT events in the evolutionary history of the CODH-hydrogenase gene cluster: (1) *cooS* and *cooF* replacement in *C. subterraneus* subsp. *tengcongensis*; (2) *cooS* and *cooF* replacement in and *C. subterraneus* subsp. *yonseiensis*; (3) transfer of the cluster as whole to a recent ancestor of *Thermoanaerobacter* sp. YS13 from a common ancestor of *C. subterraneus* subsp. *pacificus* and *C. subterraneus* subsp. *tengcongensis*; (4) *cooS. cooF*, and *cooC* transfer from *C. subterraneus* subsp. *tengcongensis* to *Thermodesulfobacterium thermophilum*.

### Other hydrogenases

Besides the CODH-associated hydrogenase, *C. subterraneus* sbsp. *tengcongensis* harbors additional hydrogenases, a NiFe hydrogenase (encoded by *ech* genes) and a NADH-dependent Fe-only hydrogenase (encoded by *hyd* genes), which putatively catalyze the production of H_2_ from excess of reducing equivalents formed during the fermentation of saccharides at low p(H_2_) [21]. The genes encoding these enzymes have been identified in *C. subterraneus* subsp. *pacificus* (although some *ech* are incomplete in the current genome assembly) and *C. subterraneus* subsp. *yonseiensis*. As pointed out by Calteau et al. [36] and Soboh et al. [21], both hydrogenase genes were wrongly assigned as NADH:ubiquinone oxidoreductase genes in *C. subterraneus* subsp. *tengcongensis* genome because of automatic annotation process. This error was also introduced to the *C. subterraneus* subsp. *yonseiensis* genome, and in the previous version of the genome of *C. subterraneus* subsp. *pacificus*. However, in the latest annotation of the genome of *C. subterraneu*s subsp. *pacificu*s, the proper description of these genes has been included.

Homologs of *E. coli hyp* genes are located upstream the *ech* genes in *C. subterraneus*. In *E. coli*, *hyp genes* are essential for the maturation of the hydrogenases [23]. The synteny of *hyp* genes adjacent to *ech* genes could indicate their role in the maturation of Ech hydrogenase. However, it is noteworthy that possibly Hyp proteins could act on this as well as other hydrogenases, notably the CODH associated hydrogenase, which resembles the *E. coli* Hyc hydrogenase (Hyd-3), target of HypA and HypC proteins [37]. In *M. thermoacetica*, the *hypABFCDE* operon, and in the *Geobacillus thermoglucosidans* strains investigated in this study, the *hypAB* genes are located downstream the *hyf*/*hyc* operon, which represents additional evidence supporting the probable interaction of their gene products.

Calteau et al. [36] had suggested that *ech* genes from an archaeon related to *Methanosarcina* were transferred horizontally to a *C. subterraneus* subsp. *tengcongensis* ancestor, however our analyses throughout parametric methods did not detect divergent sequence patterns in these genes (Additional file 2: Table S1). On the other hand, our phylogenetic analyses for most *ech* genes (with the exception of *ech*B) demonstrated that *C. subterraneus* are immersed in the *Thermoanaerobacter* clade (Additional file 5: Figure S4). Consequently, the *C. subterraneus* subspecies last common ancestor would not have acquired the *ech* genes directly from an archaeon, but more likely indirectly through a *Thermoanaerobacter* species. Therefore, the alternative hypothesis by Calteau et al. [36] would be in agreement with our observations suggesting an initial transfer of these genes from an archaeon to a bacterial lineage followed by a second bacterium to bacterium transference. The *hyp* and *hyd* genes showed characteristics expected for this species in accordance to sequence composition (Additional file 2: Table S1) and phylogeny (Additional file 6: Figure S5 and Additional file 7: Figure S6).

*E. coli* has multiple hydrogenases that act differently depending on carbon source availability and on pH [38]. Similarly, hydrogenases from *C. subterraneus* are expected to be active under different environmental conditions which would increase fitness in a variety of extreme environmental situations and carbon sources that *C. subterraneus* subspecies encounter in their natural niches [8].

### The Mbx ferredoxin:NADP oxidoreductase

**Fig. 5:**
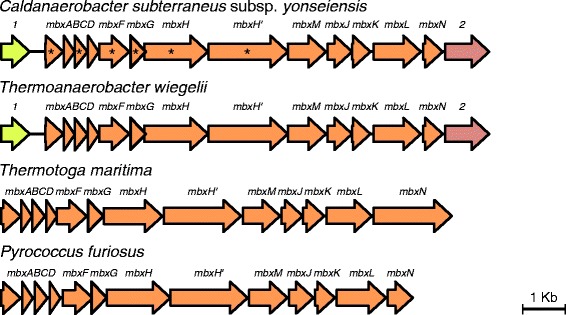
Comparison of the Mbx gene cluster organization across different species. *Arrows* represent genes and their respective direction of transcription. *Asterisks* indicate significant prediction of HGT in *C. subterraneus* subsp. *yonseiensis. 1*–putative G-D-S-L family lipolytic protein gene; *2*–putative cation transporter gene

From all *C. subterraneus* subspecies investigated in this study, *C. subterraneus* subsp. *yonseiensis* uniquely encodes an *mbx* gene cluster (genes O163_11500 to O163_11560). Its products are highly similar to *Pyrococcus furiosus* Mbx proteins (identities ranging from 30 to 60 %), which were automatically misannotated as NADH-ubiquinone oxidoreductase subunits and were initially described as encoding a putative fourth hydrogenase in *P. furiosus* [39]. However, according to the currently prevailing views, substantiated by the Adams lab [40, 41], Mbx is not a hydrogenase but a ferredoxin:NADP oxidoreductase, one of the differentiating features being the lack of the two CxxC Ni-binding motifs characteristic of [NiFe]-hydrogenases in the MbxL (HyfG) subunit (including O163_11555 in *C. subterraneus* subsp. *yonseiensis*). The genes O163_11495 and O163_11565 that flank the *mbx* operon in *C. subterraneus* subsp. *yonseiensis* were also found in the other *C. subterraneus* genomes (Fig. 5). The gene O163_11495 encodes a putative G-D-S-L family lipolytic protein (Additional file 8: Table S3), therefore it does not seem to be functionally related to Mbx hydrogenase, and the gene O163_11565 encodes for a putative cation transporter. Preliminary blast searches using the nucleotide region spanning from the gene O163_11495 to the gene O163_11565 revealed 97 % identity to a genomic region from *Thermoanaerobacter wiegelii*. Although these organisms are rather closely related (Fig. 1), the high identity between these genome fragments is unexpected (the ANI between their genomes is 82 %) and represents a strong indication of HGT. In fact, as demonstrated in Fig. 5 and Additional file 2: Table S1, some of the *mbx* genes show differential tetranucleotide composition in *C. subterraneus* subsp. *yonseiensis*. Phylogenetic analyses of each deduced Mbx protein corroborated the hypothesis that most *mbx* genes in *C. subterraneus* subsp. *yonseiensis* grouped with *Thermoanaerobacter* species (Additional file 9: Figure S7). Furthermore, in the phylogenetic reconstruction of the gene O163_11495, *C. subterraneus* subsp. *yonseiensis* is closer to *Thermoanaerobacter* than to other *C. subterraneus subspecies*, and in the phylogeny of the gene O163_11565, homologs from all *C. subterraneus* subspecies are immersed in a *Thermoanaerobacter* species clade (Additional file 9: Figure S7).

Evidence suggests that the *mbx* operon could have been transferred from a *Thermoanaerobacter* species to *C. subterraneus* subsp. *yonseiensis*. Calteau et al. [36] suggested that the *mbx* genes could have been originally transferred from Archaea to Bacteria. As the case of *ech* genes, this evolutionary event would have preceded the bacteria-to-bacteria HGTs.

### Glycosidases

Glycosidases from thermophiles have many industrial and biotechnological applications [42], thus the wealth of glycosidases in these species motivates detailed study. *C. subterraneus* subsp. *tengcongensis* presents 25 glycosidases distributed in 13 families, while *C. subterraneus* subsp. *yonseiensis* harbors 17 glycosidases from 8 families, and most of which have homologous counterparts in *C. subterraneus* subsp. *tengcongensis. C. subterraneus* subsp. *pacificus* have 21 glycosidases from 12 families, and two of them are specific to this subspecies (Additional file 2: Table S1 and Additional file 10: Table S2).

At this time three glycoside hydrolases deduced from *C. subterraneus* subsp. *tengcongensis* genome have been biochemically characterized. Two of them are starch-hydrolyzing enzymes, a glucoamylase (TTE1813) [43] and an alphaglucosidase (TTE0006) [15], and both have homologs in the other two *C. subterrraneus* subspecies (Additional file 10: Table S2). Exoglucohydrolases similar to these ones are extensively utilized for the hydrolysis of starch to glucose in industrial processes for food and ethanol production [15, 43]. Additional alphaglucosidases from the GH31 family remaining to be investigated were found in the genomes of *C. subterraneus* subsp. *tengcongensis* (TTE1934) and *C. subterraneus* subsp. *pacificus* (CDSM653_01802) (Additional file 10: Table S2). These orthologs have 32 % identity with the protein MalA of the archaeon *Sulfolobus solfataricus*, which has a substrate preference for maltose and maltooligosaccharides [44]. Their neighbor genes are sugar permease genes in both *C. subterraneus* genomes, and they present different tetranucleotide composition, suggesting a likely horizontal inheritance for these genes (Additional file 2: Table S1).

The third type of identified glycosidase is a cellulase (endoglucanase) (TTE0359) [45], which was also found in *C. subterraneus* subsp. *yonseiensis* (Additional file 10: Table S2). This enzyme is able to break the internal bonds of cellulose, generating glucans of different lengths that are substrate for other enzymes to glucose production. One of these enzymes is the betaglucosidase, which hydrolyzes cellobiose disaccharides to glucose. Putative betaglucosidases from families GH1 and GH3 were found in the genomes of *C. subterraneus* subsp. *tengcongensis* and *C. subterraneus* subsp. *pacificus*, but the genome of *C. subterraneus* subsp. *yonseiensis* only hosts one belonging to the GH3 family (Additional file 10: Table S2). Currently, intensive studies of such enzymes are being carried out, due to their implication in the saccharification of lignocellulosic materials such as sugarcane bagasse for production of biofuel [46]. Also, it is worth noting that four putative enzymes originally annotated as hypothetical in *C. subterraneus* subsp. *tengcongensis* genome belong by similarity to the GH18 family of glycosidases, and they were found in the other two *C. subterraneus* genomes (Additional file 10: Table S2). This family is known by containing chitinases, enzymes that hydrolyze chitin, one of the most common biopolymers in nature [47]. Bacterial chitinases can be utilized as biological control of fungi and insects, but are also suitable for protoplast generation and the treatment of shellfish waste [48].

### Esterases

Esterases are widely utilized in industry for production of pharmaceuticals, detergents, biodiesel and other compounds [49, 50]. At least five esterases of *C. subterraneus* subsp. *tengcongensis* have been biochemically characterized [1, 2, 51–54]. These enzymes share high thermal stability at temperatures above 60 °C, and they use different substrates, as mentioned in Additional file 8: Table S3. Besides these esterases, Levisson et al. [55] detected through *in silico* approaches four additional esterases in *C. subterraneus* subsp. *tengcongensis* genome (Additional file 8: Table S3). In our study, it was verified that *C. subterraneus* subsp. *yonseiensis* possesses homologs of each one of the esterases referenced above. Two of them were not located in *C. subterraneus* subsp. *pacificus* genome, however using the LIPABASE proteins as reference, a specific lipase was found (CDSM653_00572). It matched a lipase from *Acinetobacter baumannii* [EMBL:A3M3C1], but because the coverage was 36 % and identity 31 %, more detailed studies are necessary to evaluate its catalytic properties.

### Proteases

Proteases are ubiquitous to all life forms, with in vivo functions ranging from protein turnover to growth substrate hydrolysis and amino acid acquisition. They have a highly diverse range of applications, such as tenderization of meat, composing detergent formulations, leather processing, molecular biology applications and peptide synthesis [56].

Around 100 proteases from 50 distinct subfamilies were found in each *C. subterraneus* genome (Additional file 11: Table S4). Among these proteins, metallo- and serineproteases were the most common. We note that the M42 subfamily proteases were originally annotated as cellulase-like proteins in *C. subterraneus* subsp. *tengcongensis* (Additional file 11: Table S4), but it is likely another case of misannotation. Dutoit et al. [57] verified experimentally that two proteins annotated as cellulases in the *Thermotoga maritima* and *Clostridium thermocellum* genomes were actually M42 aminopeptidases.

Although *C. subterraneus* possesses many proteases, only one peptidase has been already characterized, a serine protease named as tengconlysin (TTE0824) [58]. Therefore the potential of *C. subterraneus* as a source for proteases is underexploited.

## Conclusions

The study of *C. subterraneus* genomes is important to understand the adaptations allowing them to thrive in extreme habitats, as well as to analyze enzymes with biotechnological potential showing functionality under high temperatures. H_2_ is an important compound to the chemical industry, and a future clean biofuel [59]. Genomic data indicate that *C. subterraneus* is able to produce H_2_ throughout different hydrogenase systems, markedly one associated with a CODH that permits obtaining energy from carbon monoxide, widely available in syngas and other industrial fuel gases. Horizontal gene transfer seems to be an important evolutionary driving force in carboxydotrophy and hydrogenogenesis in this species, abilities that permitted it to survive in niches where multiple inorganic and organic substrates may be available at low concentrations. In this sense, it is also worth noting that these bacteria encode a wide repertoire of hydrolase genes, such as glycosidases, esterases and proteases that act on a wide variety of substrates to provide them with carbon and energy. Therefore, the metabolic versatility of this species makes it a good source to target for novel enzymes with biotechnological potential.

## Methods

### Bacterial strain, genome sequencing, and operon prediction

*C. subterraneus* subsp. *pacificus* was isolated from a submarine hot vent in Okinawa Trough [8]. Genome DNA was mainly sequenced and assembled at the J. Craig Venter Institute. Contigs of *C. subterraneus* subp. *pacificus* genome were automatically annotated with the xBase platform [60] using as reference the *C. subterraneus* subsp. *tengcongensis* genome. Genes of interest were inspected carefully and had their annotation refined manually. Operons were predicted using DOOR software [61]. This Whole Genome Shotgun project has been deposited at DDBJ/EMBL/GenBank under the accession ABXP00000000. The version described in this paper is version ABXP02000000.

### 16S rRNA gene phylogeny

Most of the 16S rRNA gene sequences were retrieved from the SILVA rRNA Database (Additional file 12: Table S5) [62]. Sequences were aligned using SINA software [63], and gap positions were removed. Phylogenetic reconstructions were performed using the Phylogeny.fr platform [64] with the maximum likelihood method implemented in the PhyML program (v3.0 aLRT) [65]. For each phylogeny, the GTR (Generalized Time Reversible) substitution model was selected assuming an estimated proportion of invariant sites and 4 gamma-distributed rate categories to account for rate heterogeneity across sites. The gamma shape parameter was estimated directly from the data. Reliability for internal branching was assessed using the aLRT (approximate Likelihood Ratio Test) [66].

### Comparison of *C. subterraneus* genomes

In addition to the genome sequenced in this study, two genomes of *C. subterraneus* are publicly available from the following subspecies: *C. subterraneus* subsp. *yonseiensis* (AXDC00000000.1) and *C. subterraneus* subsp. *tengcongensis* (NC_003869.1). Only the *C. subterraneus* subsp. *tengcongensis* genome is complete. Therefore, the following comparative analyses were made using this genome as reference.

The average nucleotide identity (ANI) values (species boundary is 95 %) for the genomes of Caldanaerobacter subterraneus subspecies were determined [67]. The in silico prediction of in vitro DNA-DNA hybridization (DDH) values (species boundary is 70 %) were calculated using GGDC 2.0 BLAST+ and recommended formula 2 [68].

Synteny plots were generated with the R2CAT software [69], aligning and ordering the contigs of *C. subterraneus* subsp. *pacificus* and *C. subterraneus* subsp. *yonseiensis* against *C. subterraneus* subsp. *tengcongensis* genome.

mGenome Subtractor [70] was utilized to compare the conservation of proteins of *C. subterraneus* subsp. *pacificus* and *C. subterraneus* subsp. *yonseiensis* genomes in relation to those from *C. subterraneus* subsp. *tengcongensis*.

The homology score (H-value) between two proteins is the product of the identity level (expressed as a value between 0 and 1) and of the ratio of the match length to query length [70]. Conserved proteins were defined by having a homology score H-value above 0.64.

### Horizontal gene transfer analysis

The genomes of *C. subterraneus* and the genomes from related species of interest were utilized for HGT detection throughout the GOHTAM platform [71], which detects horizontal gene transfers based on tetranucleotide composition and/or codon usage. GC content of the CDSs was computed using EMBOSS package [72, 73]. Genes containing a GC content higher or lower than two standard deviations from the average CDS GC content for all CDSs in a genome were highlighted.

### Other phylogenetic analyses

Amino acid sequences of *Caldanaerobacter subterraneus* subspecies were utilized as query in blastp searches against the Genbank NR database. The most similar sequences were retrieved. Also, for some proteins (e.g. CooS), homologous counterparts from PDB database were also retrieved. Subsequently, amino acid sequences were aligned using MUSCLE software embedded in MEGA [74]. Sites from the alignment containing gaps were removed. The phylogeny was constructed on the Phylogeny.fr platform basically as described previously above, but this time the WAG substitution model [75] was utilized.

### Genome mining for hydrolases

Predicted proteins of the three subspecies of *C. subterraneus* were used as queries in blastp searches for glycohydrolase, esterase, peptidase and lipase databases. For protease identification the batch blast tool from MEROPS database [76] was utilized. For each protein, the hit with the lowest e-value (<10^−10^) was considered. Glycoside hydrolases were identified using CAT (Cazymes Analysis Tool) [77] with the following parameters: complete genome and e-value threshold of 10^−10^. Only glycosidases with domain and length consistency were considered. Esterases homologous to those already identified in *C. subterraneus* subsp. *tengcongesis* [1, 2, 51–55] were identified in the other subspecies genomes using as criterion the H-value >0.64. Lipases were searched among the *C. subterraneus* proteins using the blastp tool embedded in Bioedit [78] against the lipase database LIPABASE [79], with an e-value cutoff set to 10^−10^.

## Availability of supporting data

Supporting data are included as Additional files. Phylogenetic data have been deposited at TreeBASE under the accession URL http://purl.org/phylo/treebase/phylows/study/TB2:S18113.

## Additional files

Additional file 1: Figure S1. Alignment of CODHs (CooS) from *C. subterraneus* with archetypical CODHs from other prokaryotes. *Cp C. subterraneus* subsp. *pacificus*, *Ct C. subterraneus* subsp. *tengcongensis* (NP_623304.1), *Cy C. subterraneus* subsp. *yonseiensis* (ERM92236.1), *Ch Carboxydothermus hydrogenoformans* (WP_011343033.1), *Mt Moorella thermoacetica* ATCC 39073 (YP_430060.1), *Rr Rhodospirillum rubrum* ATCC_11170 (YP_426515.1). Black boxes represent 100 % identity. Purple letters–Cluster C, red letters–Cluster B and green letters–Cluster D, as defined by Dobbek et al. [80]. (PDF 33 kb) Additional file 2: Table S1. Genome analyses of *C. subterraneus* subspecies and other bacteria. (XLSX 965 kb) Additional file 3: Figure S2. Evolutive history of CooC and CooF proteins from *C. subterraneus* subspecies. The tree was constructed using the maximum-likelihood method. aLRT values greater than 70 % are shown next to the branches. The tree is drawn to scale, with branch lengths in the same units as those of the evolutionary distances used to infer the phylogenetic tree. *Caldanaerobacter subterraneus* subspecies are in bold. Other Clostridiales species are in green, Bacillales in blue, Proteobacteria in purple, Thermodesulfobacteria in orange, Archaea in red, and other bacteria in black. Accession number or locus tag are adjacent the species name. The tree is mid-point rooted. (PDF 57 kb) Additional file 4: Figure S3. Evolutive history of Hyc and Hyf proteins from *Caldanaerobacter subterraneus* subspecies. The subtrees were extracted from trees constructed using the maximum-likelihood method. Details are as shown in Additional file 3: Figure S2, unless specified otherwise. (PDF 59 kb) Additional file 5: Figure S4. Evolutive history of Ech hydrogenase from *C. subterraneus* subspecies. The subtrees were extracted from trees constructed using the maximum-likelihood method. Details are as shown in Additional file 3: Figure S2, unless specified otherwise. (PDF 55 kb) Additional file 6: Figure S5. Evolutive history of Hyp proteins from *C. subterraneus* subspecies. The subtrees were extracted from trees constructed using the maximum-likelihood method. Details are as shown in Additional file 3: Figure S2, unless specified otherwise. (PDF 58 kb) Additional file 7: Figure S6. Evolutive history of Hyd proteins from *C. subterraneus* subspecies. The subtrees were extracted from trees constructed using the maximum-likelihood method. Details are as shown in Additional file 3: Figure S2, unless specified otherwise. (PDF 56 kb) Additional file 8: Table S3. Esterases found in *C. subterraneus* genomes. (XLSX 7 kb) Additional file 9: Figure S7. Evolutive history of Mbx from *C. subterraneus subsp. yonseiensis*. Details are as shown in Additional file 3: Figure S2, unless specified otherwise. (PDF 70 kb) Additional file 10: Table S2. Glycohydrolases found in *C. subterraneus* genomes. (XLSX 7 kb) Additional file 11: Table S4. Proteases found in *C. subterraneus* genomes. (XLSX 11 kb) Additional file 12: Table S5. 16S rRNA sequences utilized in this study. (XLSX 11 kb)
