# Supplementary material for: Analysis of three genomes within the thermophilic bacterial species Caldanaerobacter subterraneus with a focus on carbon monoxide dehydrogenase evolution and hydrolase diversity
Source: BMC Genomics. 2015 Oct 7;16:757. doi: 10.1186/s12864-015-1955-9 (PMC4596419; doi:10.1186/s12864-015-1955-9)
Supplement: Additional file 1: Figure S1. — Alignment of CODHs (CooS) from C. subterraneus with archetypical CODHs from other prokaryotes. Cp C. subterraneus subsp. pacificus, Ct C. subterraneus subsp. tengcongensis (NP_623304.1), Cy C. subterraneus subsp. yonseiensis (ERM92236.1), Ch Carboxydothermus hydrogenoformans (WP_011343033.1), Mt Moorella thermoacetica ATCC 39073 (YP_430060.1), Rr Rhodospirillum rubrum ATCC_11170 (YP_426515.1). Black boxes represent 100 % identity. Purple letters–Cluster C, red letters–Cluster B and green letters–Cluster D, as defined by Dobbek et al. [80]. (PDF 33 kb) [file 12864_2015_1955_MOESM1_ESM.pdf]

```
              *         20         *         40         *         60         *         80         *        100
Cp: ----------------------MKNVHSIDPAVTKMLEIARKQGMETAWDRYLAQKPQCGFGELGICCRNCNMGPCRIDPFGEGPQKGVCGATADTIVAR
Ct: --------------------MTKQCKVSLDPAVCEMVEKARRVKVETVWDRYQAMLPQCGFGETGLCCRHCLQGPCRIDPFGGEPKLGICGATADVIVAR
Cy: --------------------MAKLSKTSLDPAVVEMVEKAKSMEIKTVWDRYQAMVPQCGFGETGLCCRHCLMGPCRIEPFGGEPKRGICGATADVIVAR
Ch: --------------------MAKQNLKSTDRAVQQMLDKAKREGIQTVWDRYEAMKPQCGFGETGLCCRHCLQGPCRINPFGDEPKVGICGATAEVIVAR
Mt: MPRFRDLSHNCRPSEAPRVMEPKNRDRTVDPAVLEMLVKSKDDKVITAFDRFVAQQPQCKIGYEGICCRFCMAGPCRIKATDGPGSRGICGASAWTIVAR
Rr: ------------------MTHHDCAHCSSDACATEMLNLAEANSIETAWHRYEKQQPQCGFGSAGLCCRICLKGPCRIDPFGEGPKYGVCGADRDTIVAR

              *        120         *        140         *        160         *        180         *        200
Cp: NLLRMIAAGASAHSDHGRDVVETLRGVGTGEAKDYTIKDEKKLMTLCKEFNITTEDKSIHEIAQELGEAALEEFGTQ--KGYIQ-TIERVPLQRKEIWKN
Ct: GLDRAIAAGAASHSGHARHLAHTLKLAAKGKARDYTIKDKAKLRSVAARLGIPTEGRSIAEIALDVAEAALADFHEK--DTPVMWAATTVTKKRARLFEE
Cy: GLDRAIAAGAAAHSGHARHLAHTLKMVAKKEARDYSIKDKNKLYKVAESLGISTEGKSEDKIALAVADAALSDFNEKPTEAPTAWVAKTVTEKRRNLLTE
Ch: GLDRSIAAGAAGHSGHAKHLAHTLKKAVQGKAASYMIKDRTKLHSIAKRLGIPTEGQKDEDIALEVAKAALADFHEK--DTPVLWVTTVLPPSRVKVLSA
Mt: NVGLMILTGAAAHCEHGNHIAHALVEMAEGKAPDYSVKDEAKLKEVCRRVGIEVEGKSVLELAQEVGEKALEDFRRLKGEGEATWLMTTINEGRKEKFRT
Rr: HLVRMIAAGTAAHSEHGRHIALAMQHISQGELHDYSIRDEAKLYAIAKTLGVATEGRGLLAIVGDLAAITLGDFQNQDYDKPCAWLAASLTPRRVKRLGD

              *        220         *        240         *        260         *        280         *        300
Cp: LGIIPRGIDREIVESLHRTHMGVDHDPASLILHGLRCSLSDGWGGSMLATEFSDVLFGTPRPIRGQQNLGVIREDAVNIVVHGHEPVLSEMLVEAVQDPE
Ct: KGLLPKGIDYEVSDIMHRTSYGVDADPVNLLLGGLRCGLAD-LAGCYMGTDMADILFGTPQPVVTEANMGVLKADAVNVAVHGHNPVLSEVIVAVAK--E
Cy: QGLMPKGIDYEISDIMHRTHMGCDADPINLLQAGIRAGLAD-LAGCTMGTEISDILFGTPQPVVSEANLGVLKADAVNVAVHGHNPVLSEIIVEVAE--D
Ch: HGLIPAGIDHEIAEIMHRTSMGCDADAQNLLLGGLRCSLAD-LAGCYMGTDLADILFGTPAPVVTESNLGVLKADAVNVAVHGHNPVLSDIIVSVSK--E
Mt: HNVVPFGIHASISELVNQAHMGMDNDPVNLVFSAIRVALAD-YTGEHIATDFSDILFGTPQPVVSEANMGVLDPDQVNFVLHGHNPLLSEIIVQAAR--E
Rr: LGLLPHNIDASVAQTMSRTHVGCDADPTNLILGGLRVAMAD-LDGSMLATELSDALFGTPQPVVSAANLGVMKRGAVNIAVNGHNPMLSDIICDVAA--D

              *        320         *        340         *        360         *        380         *        400
Cp: LEALAKKNGAS-GINLVGMCCTGNEILMRHGIPSAGNFLHQELAVMTGAIEAMVVDVQCIMPSLAQLASCYHTKFISTSPKAKFPGALHIQFEEHHALDV
Ct: MEAEAKAAGAS-GINVVGICCTGNEVLMRHGIPACTHSVSQEMALVTGALDAMVVDYQCIMPSLATVAECMGTKLITTMEIAKIPGAIHIEFSEEKAGEK
Cy: MKGEAQAVGAK-GVNVVGICCTGNEVLMRHGIPSCTHSLSQEMALITGALDAVVMDYQCIYPSLATVAECMGTKLITTMETMKLPGAIHIPFEEKRAKDI
Ch: MENEARAAGAT-GINVVGICCTGNEVLMRHGIPACTHSVSQEMAMITGALDAMILDYQCIQPSVATIAECTGTTVITTMEMSKITGATHVNFAEEAAVEN
Mt: MEGEAKAAGAK-GINLVGICCTGNEVLMRQGIPLVTSFASQELAICTGAIDAMCVDVQCIMPSISAVAECYHTRIITTADNAKIPGAYHIDYQTATAIES
Rr: LRDEAIAAGAAEGINIIGICCTGHEVMMRHGVPLATNYLSQELPILTGALEAMVVDVQCIMPSLPRIAECFHTQIITTDKHNKISGATHVPFDEHKAVET

              *        420         *        440         *        460         *        480         *        500
Cp: AREIVRQAIENYPHRN--KSRVFIPKETAEYVGGFSVEAILEALGGTWT-----PLIEAIKSKKIRGIAALVGCNNPKVTHDYNHVNMTKALIAQDVLVV
Ct: AREILRLAIETFTRRR--GKPVDIPPYKTKVVAGFSVEAIVKALSKLNAEDPLKPLIDQIAAGNIRGVCLFAGCNNVKVPQDQNFTAIARRLLKENVLVL
Cy: AREIIRQAIQAFTQRR--GKPVDIPQVKHRVVAGFSVEAIVNALSKLNAEDPLKPLIDNIVSGNLRGVCLFCGCNNVKVPQDRNFTIMVRRMLKENVLVI
Ch: AKQIIRLAIDTFKRRK--GKPVEIPNIKTKVVAGFSTEAIINALSKLNANDPLKPLIDNVVNGNIRGVCLFAGCNNVKVPQDQNFTTIARKLLKQNVLVV
Mt: AKTAIRMAIEAFKERKESNRPVYIPQIKNRVVAGWSLEALTKLLATQNAQNPIRVLNQAILDGELAGVALICGCNNLKGFQDNSHLTVMKELLKNNVFVV
Rr: AKTIIRMAIAAFGRRD--PNRVAIPAFKQKSIVGFSAEAVVAALAKVNADDPLKPLVDNVVNGNIQGIVLFVGCNTTKVQQDSAYVDLAKSLAKRNVLVL

              *        520         *        540         *        560         *        580         *        600
Cp: ETGCAAIASAKAGLLLPETA-QLAGSGLASVCRTLG--------IPPVLHMGSCVDISRILVMAAAIAKELGVDIADLPIAGAAPEWMSEKAVSIGAYVV
Ct: ATGCGAGALMRHGFMDPANVGELCGEGLKAVLTAIGEANGLGGPLPPVLHVGSCVDNSRAVALAVAVADRLGVDTDQLPVVASAAEAVAEKAVSIGTYAV
Cy: ASGCGAGALMRHGFMDPSRTKEVCGEGLKAVLTAIGEANGLGEPLPPVLHIGSCVDTSRAVALATALGNRLGVDIDQLPVVASAPEAMAEKAVAIGTWAV
Ch: ATGCGAGALMRHGFMDPANVDELCGDGLKAVLTAIGEANGLGGPLPPVLHMGSCVDNSRAVALVAALANRLGVDLDRLPVVASAAEAMHEKAVAIGTWAV
Mt: ATGCSAQAAGKLGLLDPANVETYCGDGLKGFLKRLGEGANIEIGLPPVEHMGSCVDNSRAVDLLMAMANDLGVDTPKVPFVASAPEAMSGKAAAIGTWWV
Rr: ATGCAAGAFAKAGLMTSEATTQYAGEGLKGVLSAIGTAAGLGGPLPLVMHMGSCVDNSRAVALATALANKLGVDLSDLPLVASAPECMSEKALAIGSWAV

              *        620         *        640         *        660         *
Cp: ASGIFTVLGTVPPVLGSKTVTEILTQKAKYLVGGYFAVEPDPFKAAELIINHIDEKRAALGI----------------
Ct: ALGLPTHVGVMLPVLGGPLVTKVLTDKVKELTGGYFIVDLDPESAAEKLLAAIDERRAALGLSVPGGGRR--------
Cy: AIGIPTHVGVPLPVVGGPLVTRVLTDRVKELTGGYFIVELDPEAAAQKLLTVIDERRAALGLSVPGGGRR--------
Ch: TIGLPTHIGVLPPITGSLPVTQILTSSVKDITGGYFIVELDPETAADKLLAAINERRAGLGLPW--------------
Mt: SLGVPTHVGTMPPVEGSDLIYSILTQIASDVYGGYFIFEMDPQVAARKILDALEYRTWKLGVHKEVAERYETKLCQGY
Rr: TIGLPTHVGSVPPVIGSQIVTKLVTETAKDLVGGYFIVDTDPKSAGDKLYAAIQERRAGLGL----------------
```
