# Supplementary figures and images for: Analysis of three genomes within the thermophilic bacterial species Caldanaerobacter subterraneus with a focus on carbon monoxide dehydrogenase evolution and hydrolase diversity
Source: BMC Genomics. 2015 Oct 7;16:757. doi: 10.1186/s12864-015-1955-9 (PMC4596419; doi:10.1186/s12864-015-1955-9)

## CooC

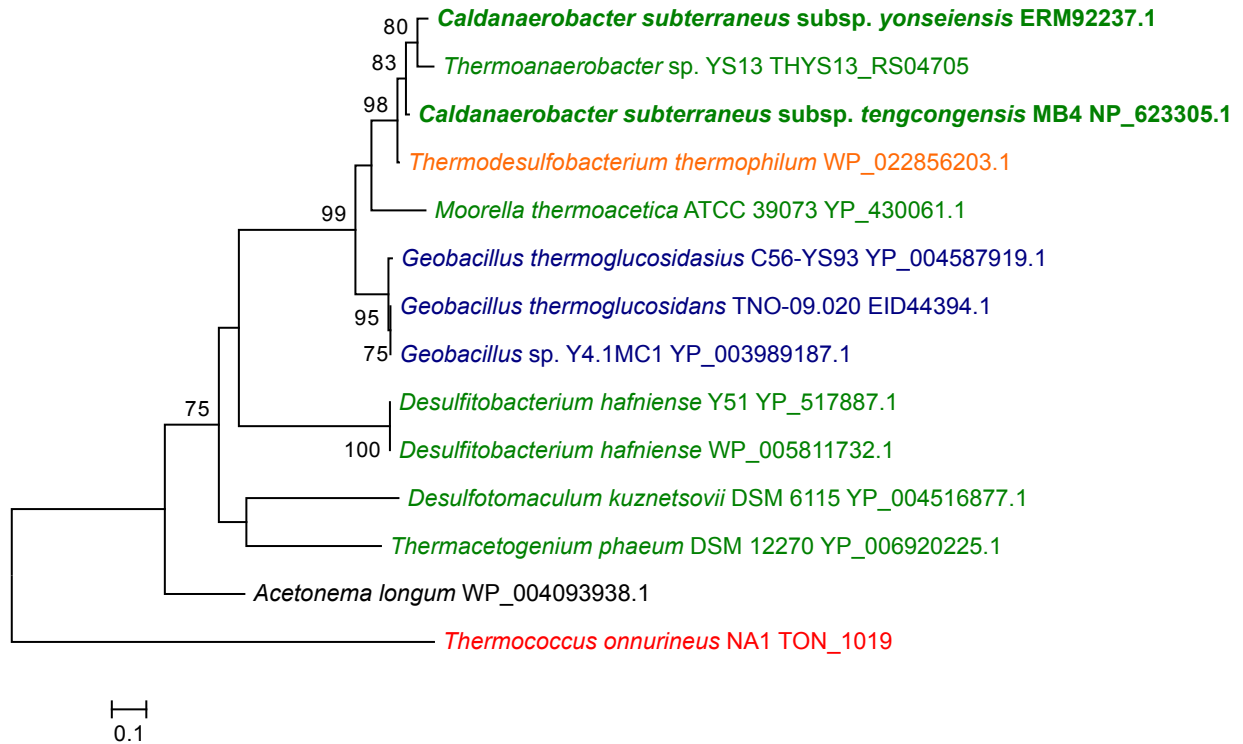

## CooF

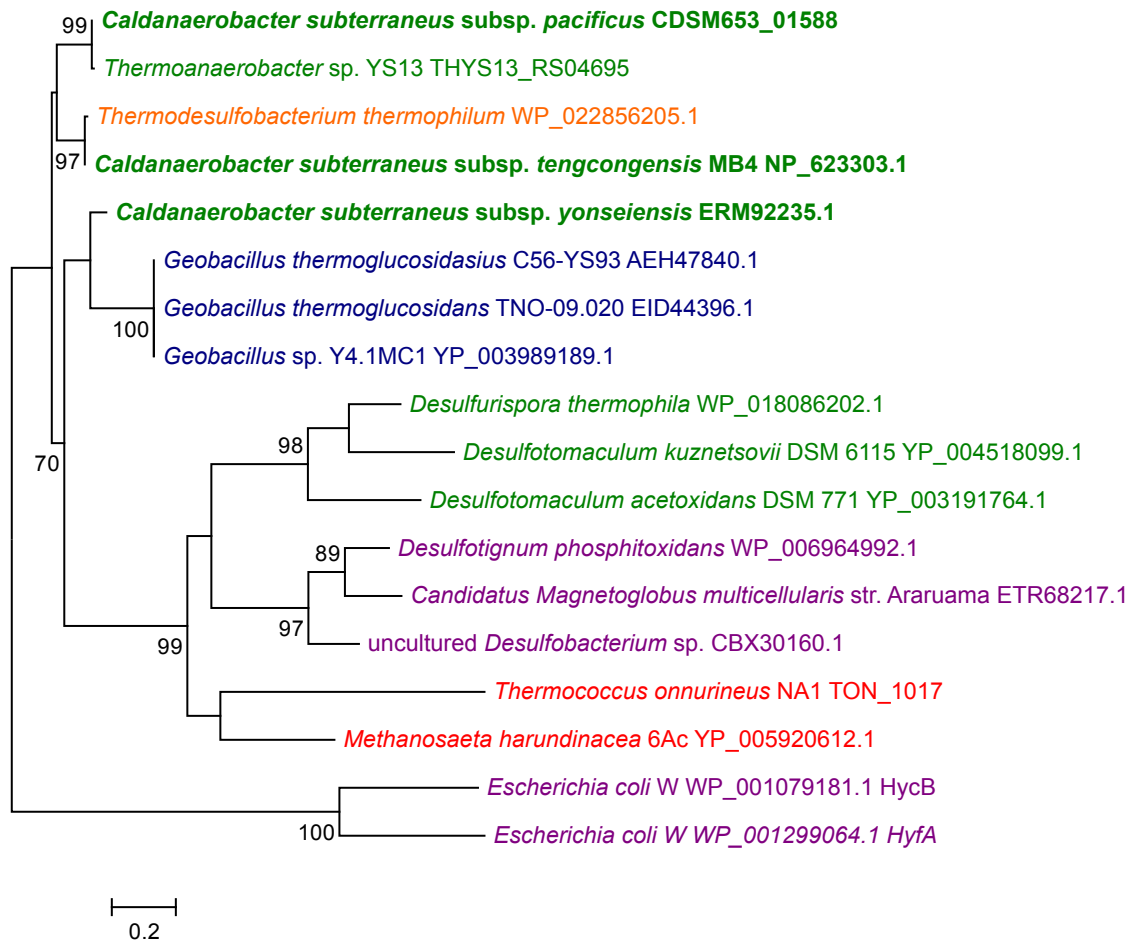

Supplement: Additional file 3: Figure S2. — Evolutive history of CooC and CooF proteins from C. subterraneus subspecies. The tree was constructed using the maximum-likelihood method. aLRT values greater than 70 % are shown next to the branches. The tree is drawn to scale, with branch lengths in the same units as those of the evolutionary distances used to infer the phylogenetic tree. Caldanaerobacter subterraneus subspecies are in bold. Other Clostridiales species are in green, Bacillales in blue, Proteobacteria in purple, Thermodesulfobacteria in orange, Archaea in red, and other bacteria in black. Accession number or locus tag are adjacent the species name. The tree is mid-point rooted. (PDF 57 kb) [file 12864_2015_1955_MOESM3_ESM.pdf]

Hych

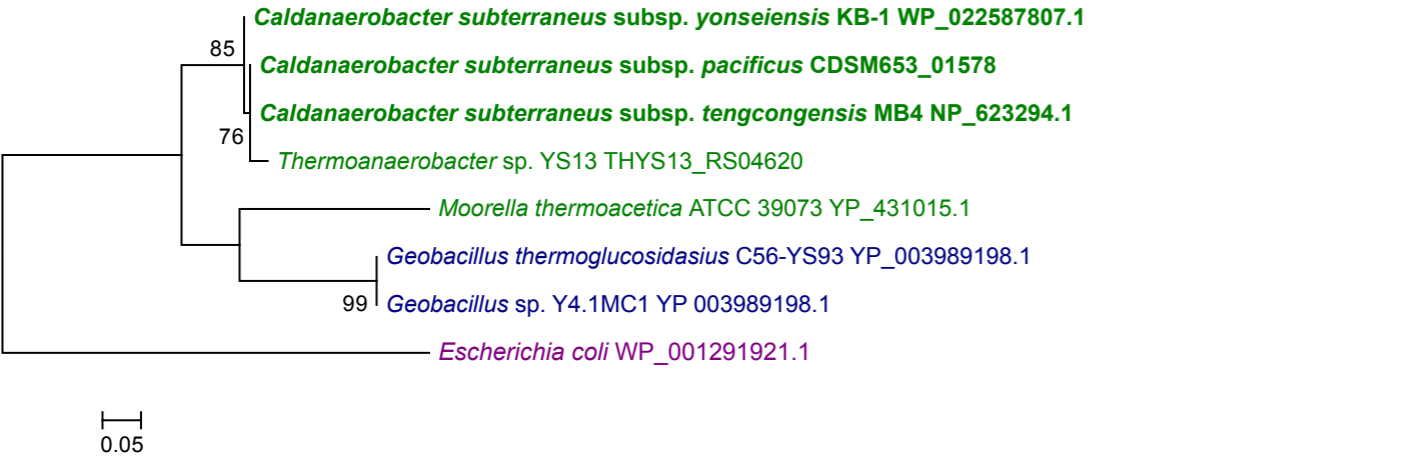

HyfE

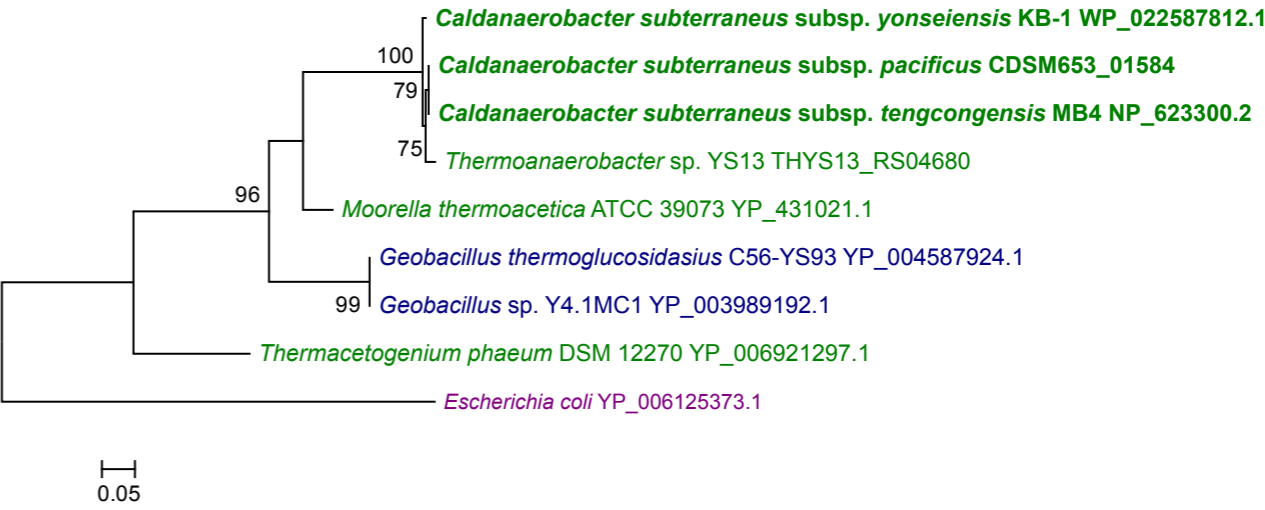

HyfI

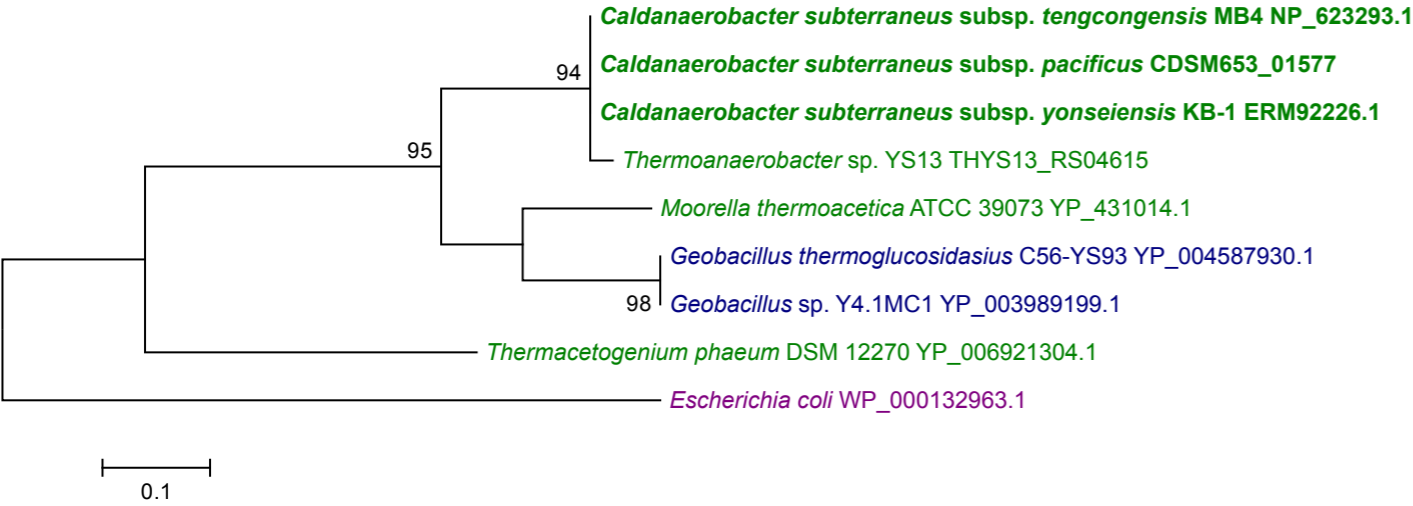

HyfF

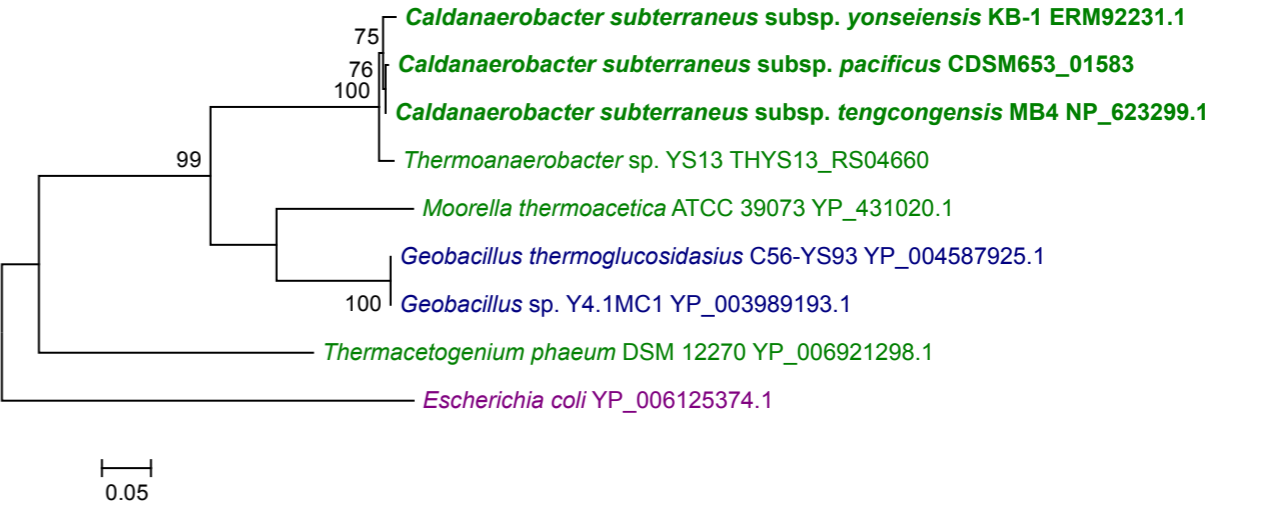

HyfB

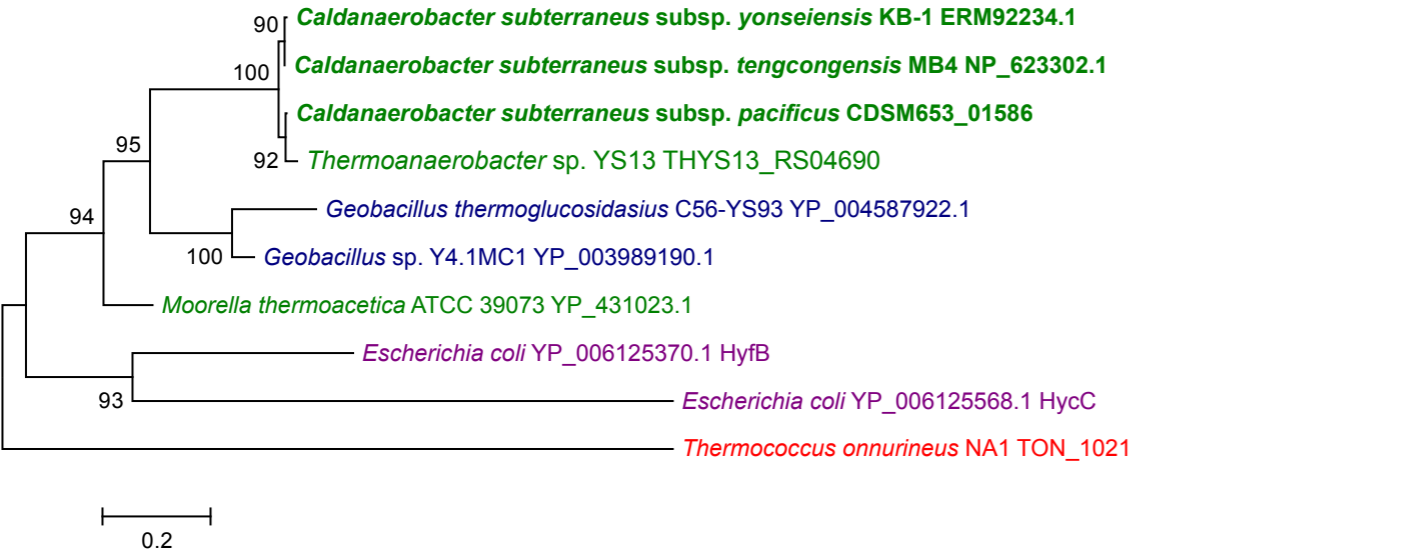

HyfG

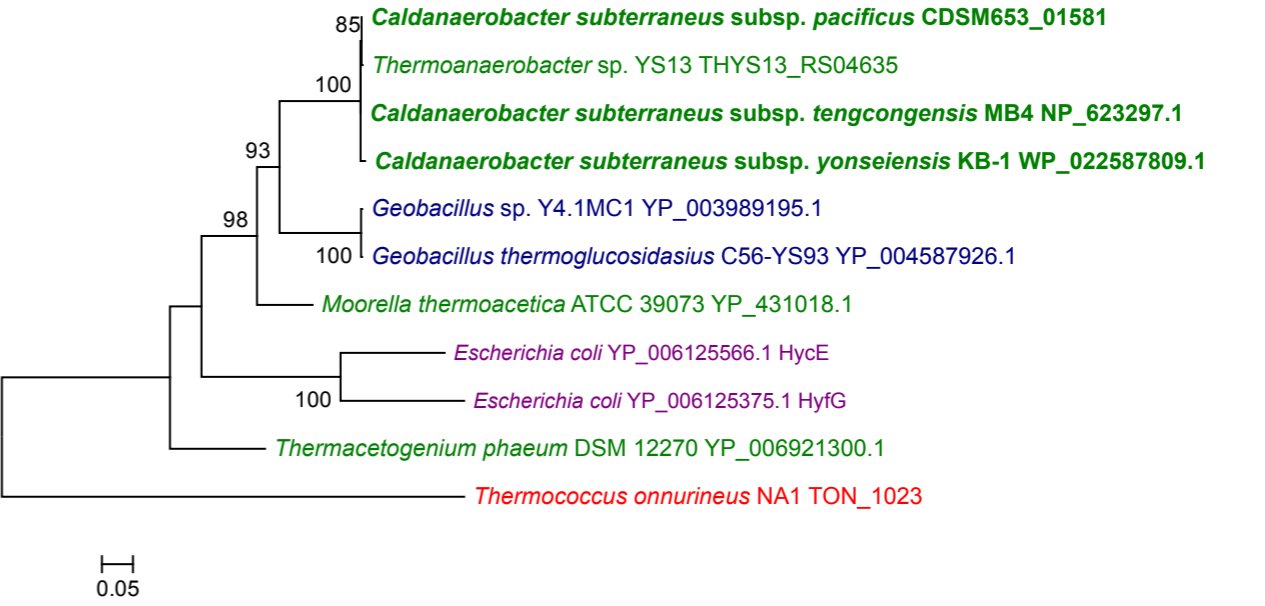

HyfC

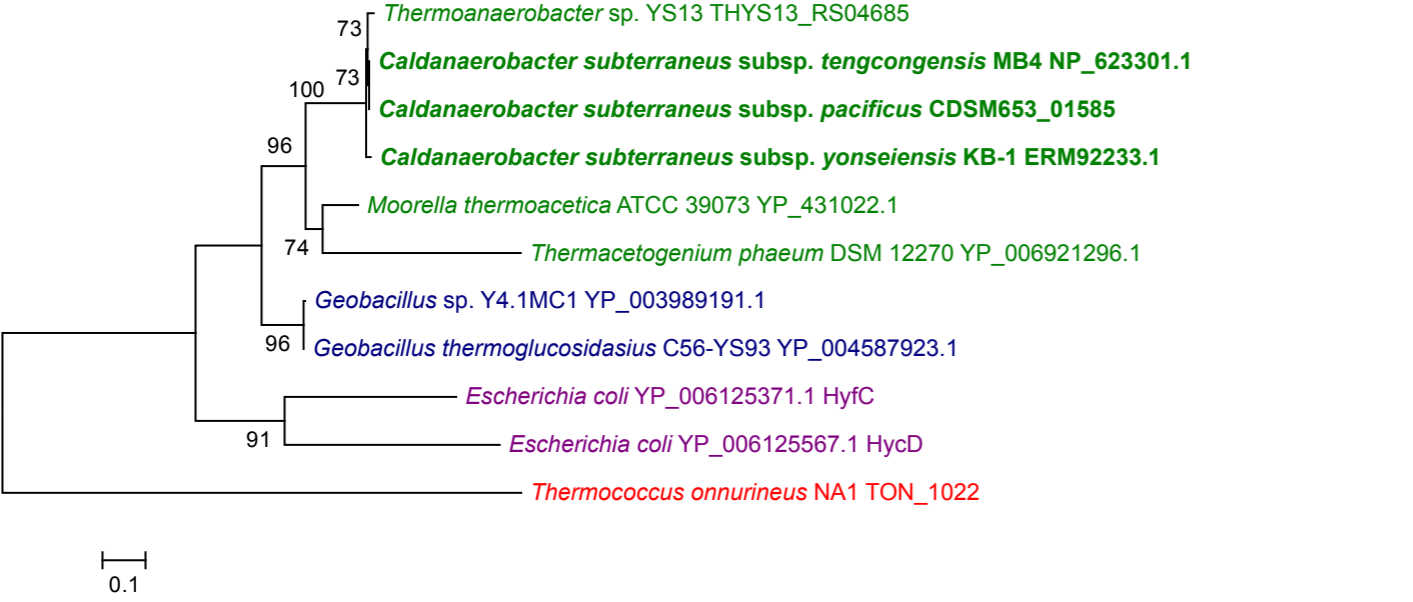

HyfH

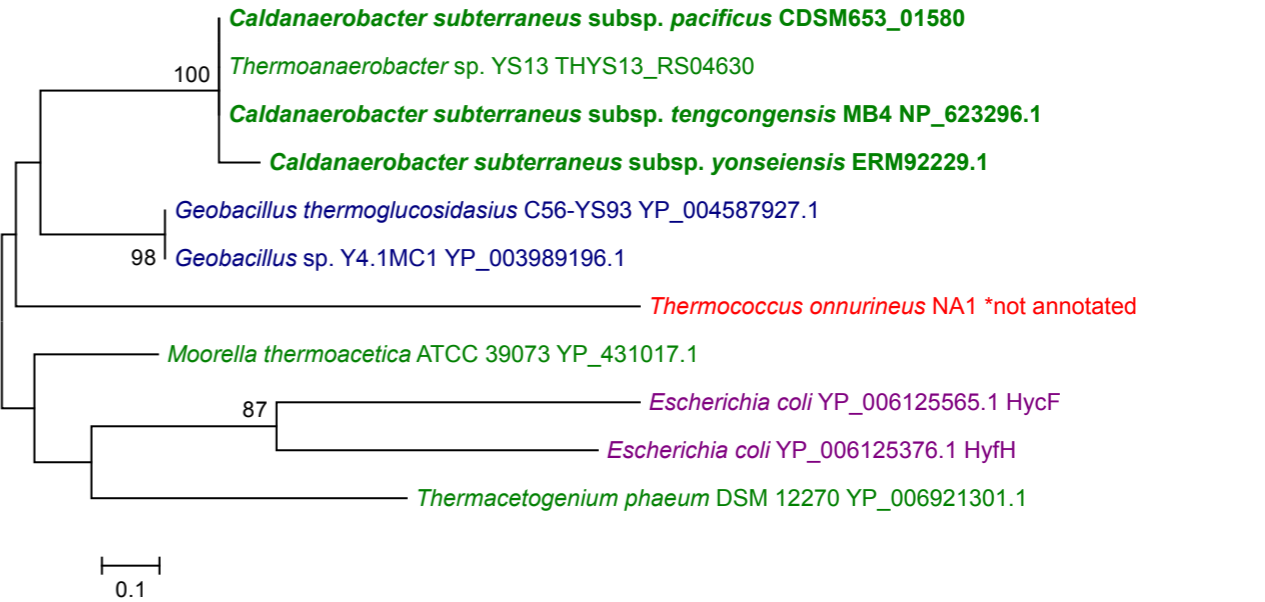

HyfD

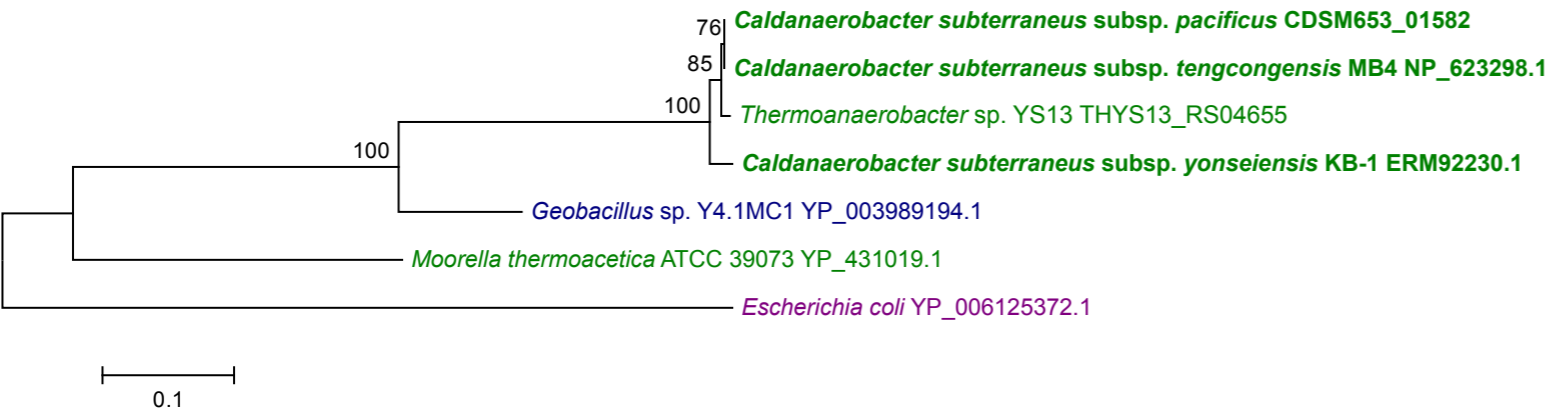

HyfI

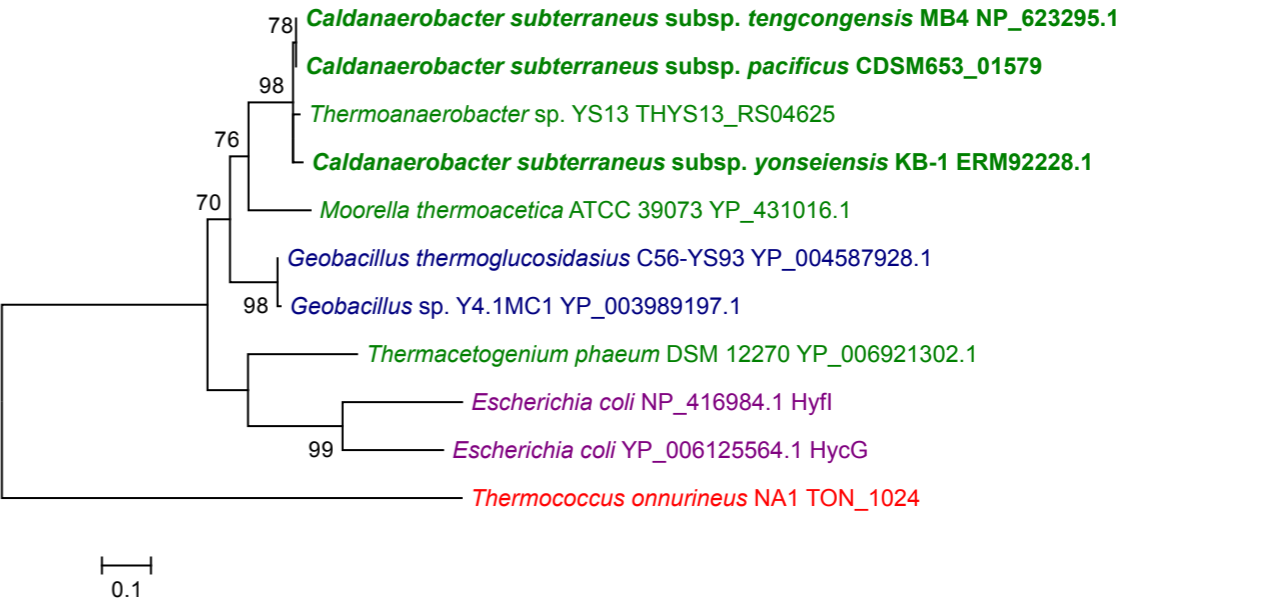

Supplement: Additional file 4: Figure S3. — Evolutive history of Hyc and Hyf proteins from Caldanaerobacter subterraneus subspecies. The subtrees were extracted from trees constructed using the maximum-likelihood method. Details are as shown in Additional file 3: Figure S2, unless specified otherwise. (PDF 59 kb) [file 12864_2015_1955_MOESM4_ESM.pdf]

EchA

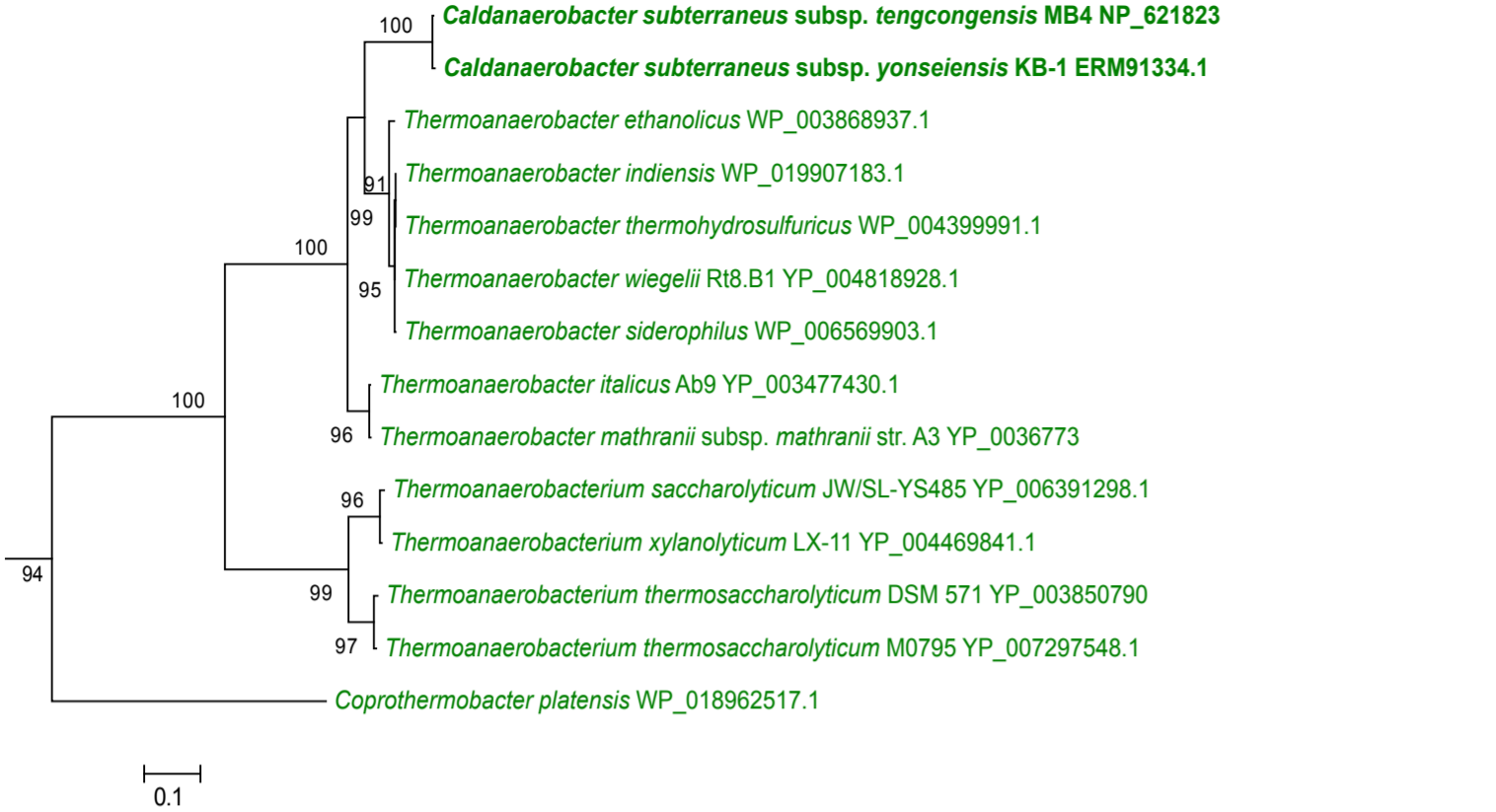

EchB

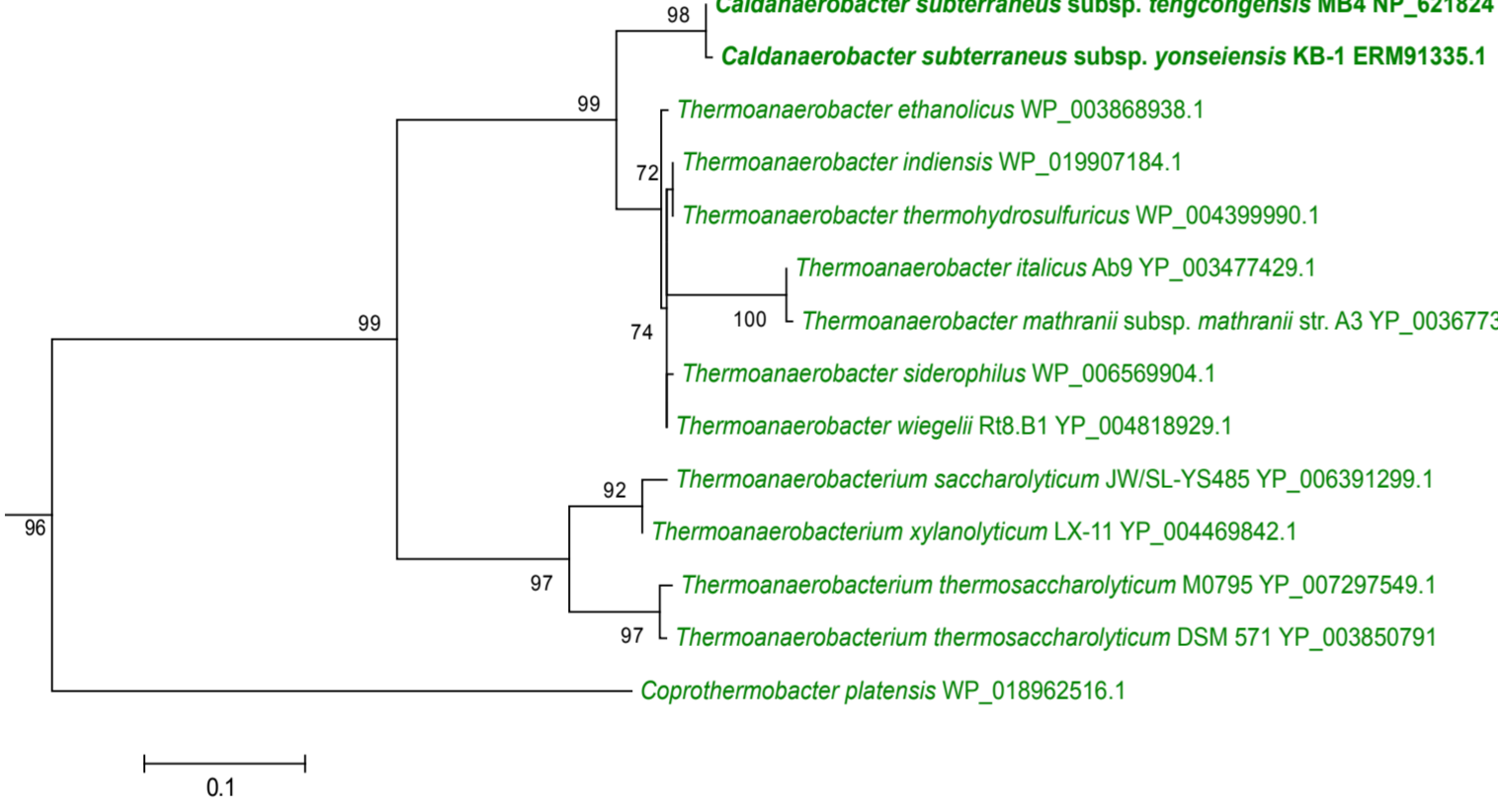

EchC

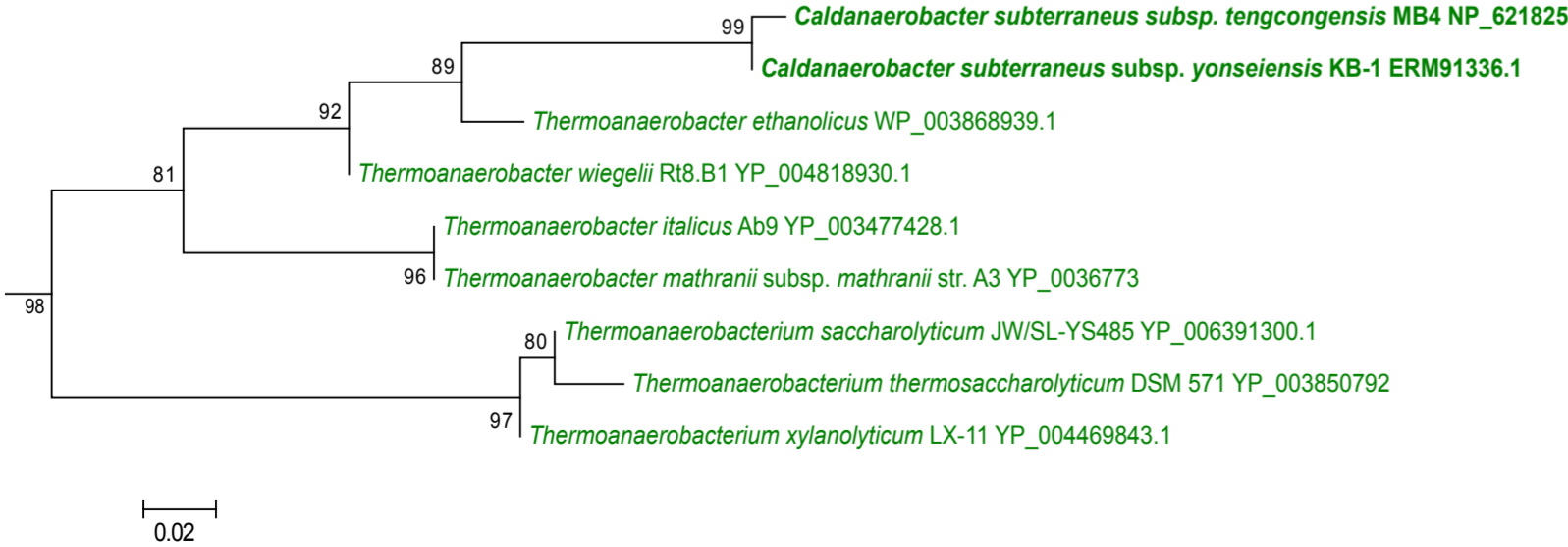

EchD

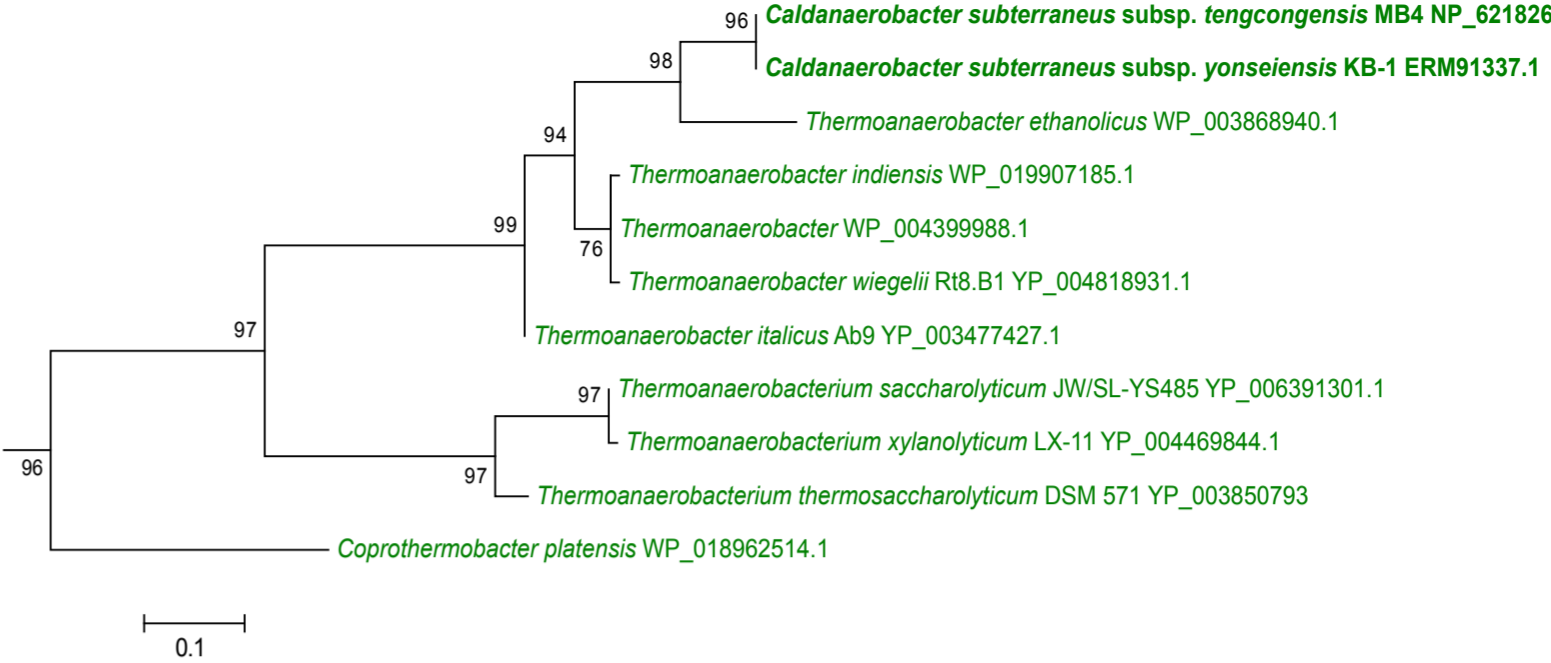

EchE

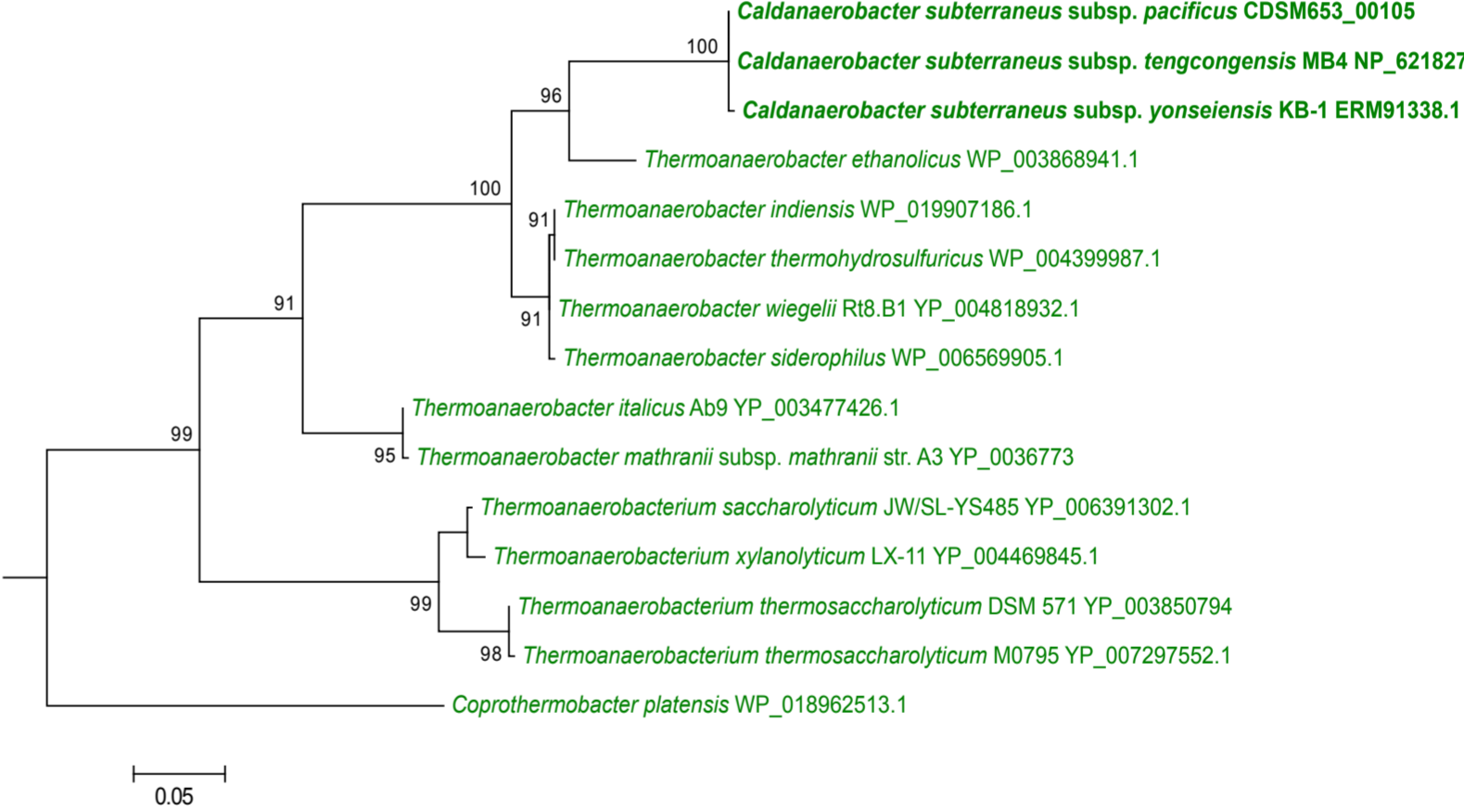

EchF

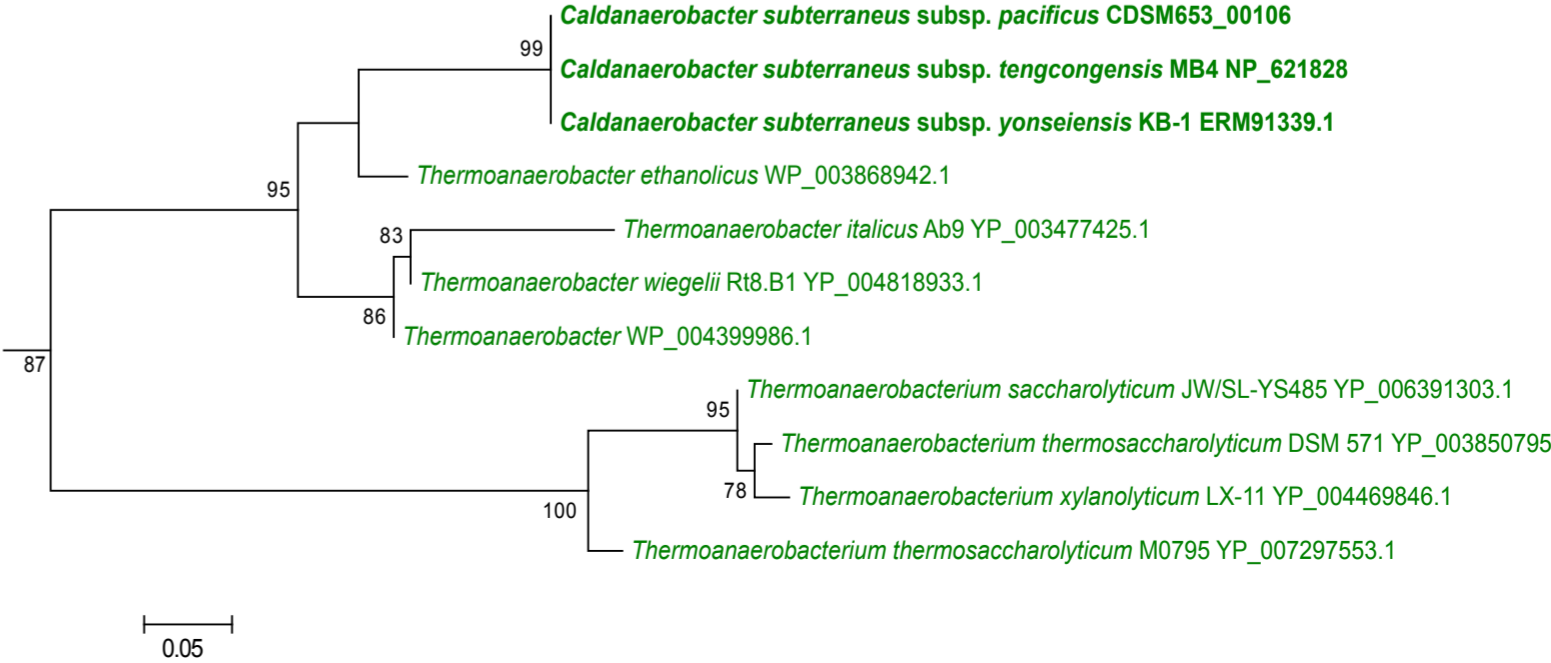

Supplement: Additional file 5: Figure S4. — Evolutive history of Ech hydrogenase from C. subterraneus subspecies. The subtrees were extracted from trees constructed using the maximum-likelihood method. Details are as shown in Additional file 3: Figure S2, unless specified otherwise. (PDF 55 kb) [file 12864_2015_1955_MOESM5_ESM.pdf]

HypA

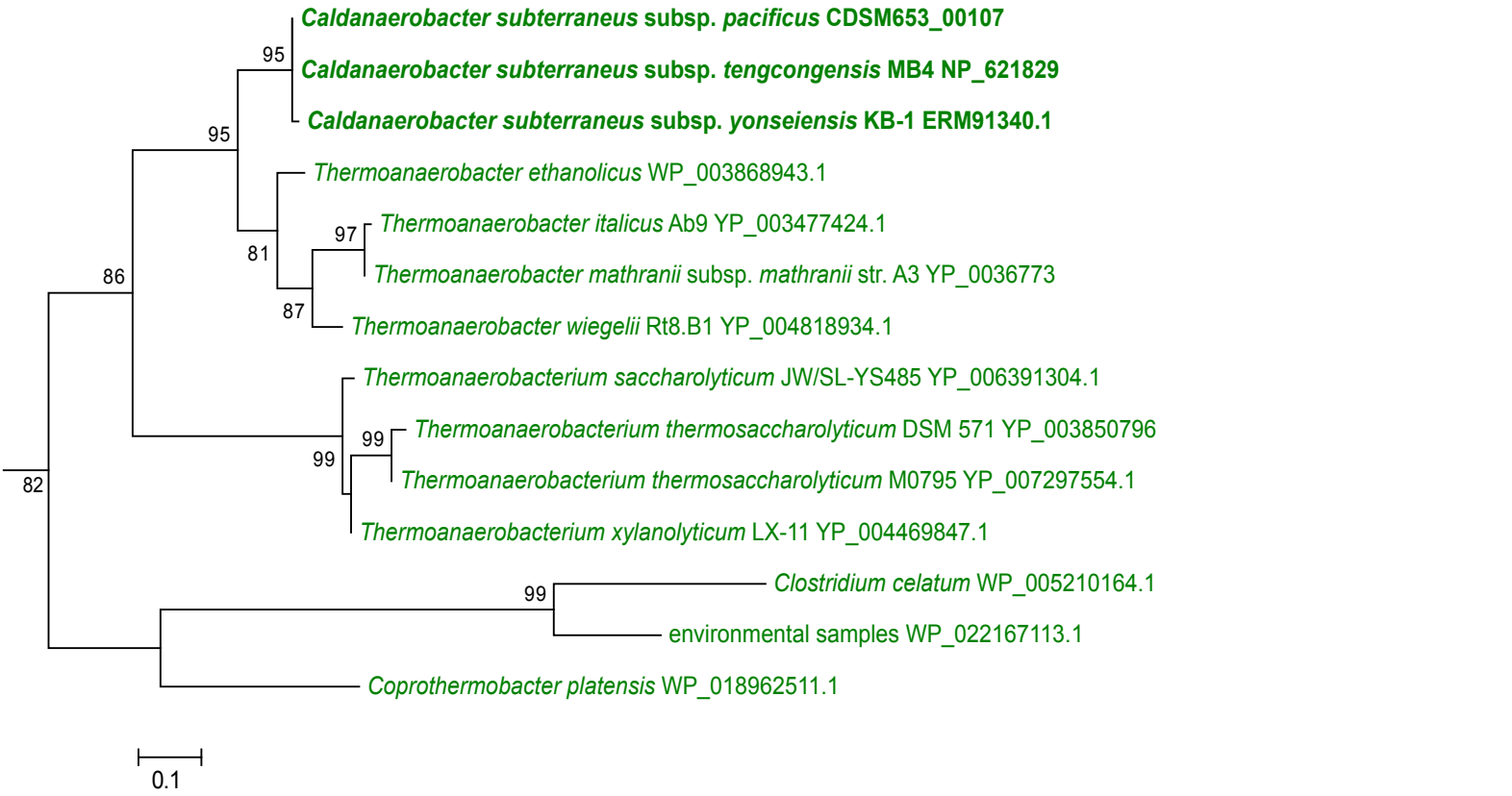

HypD

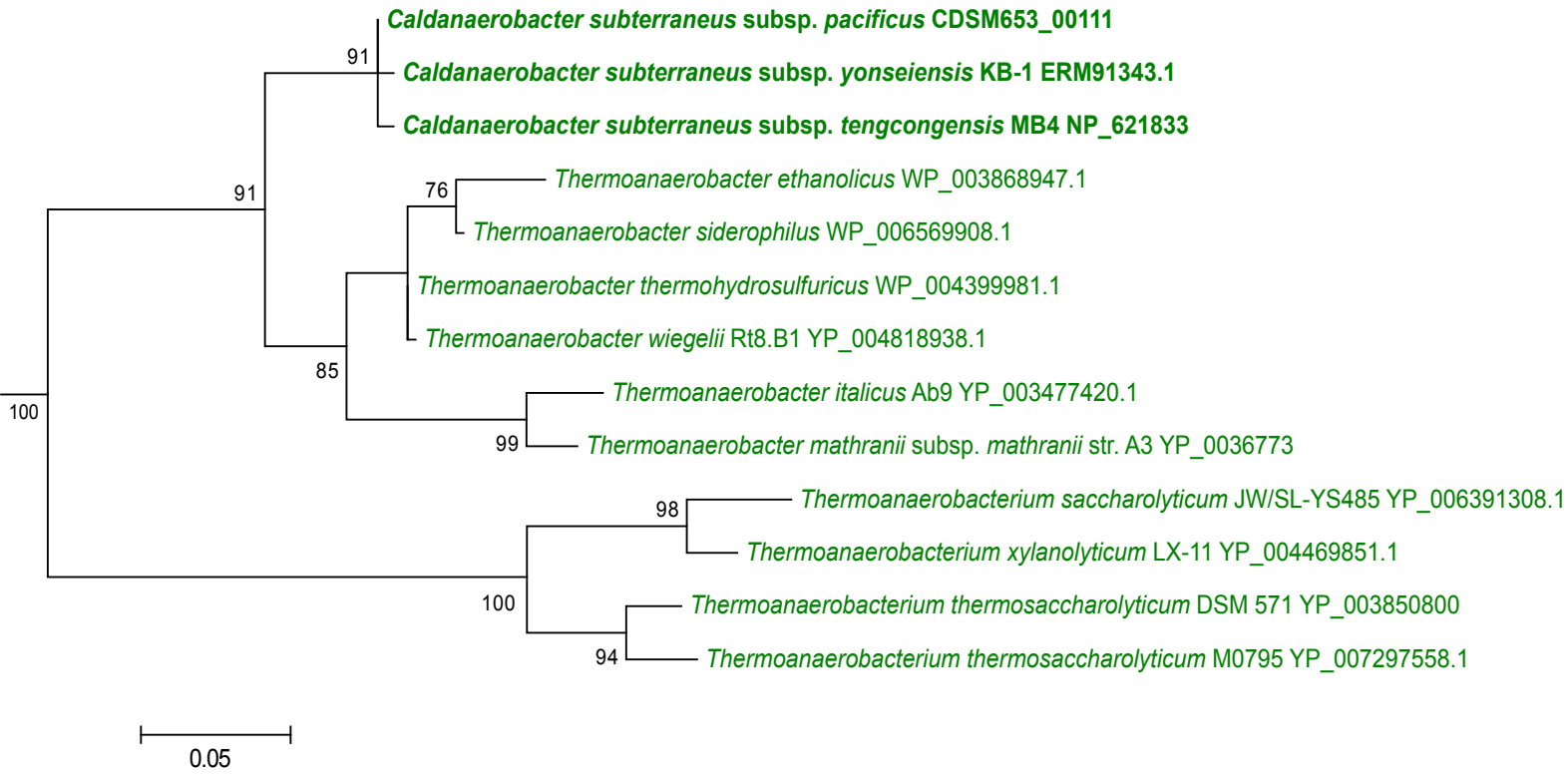

HypB

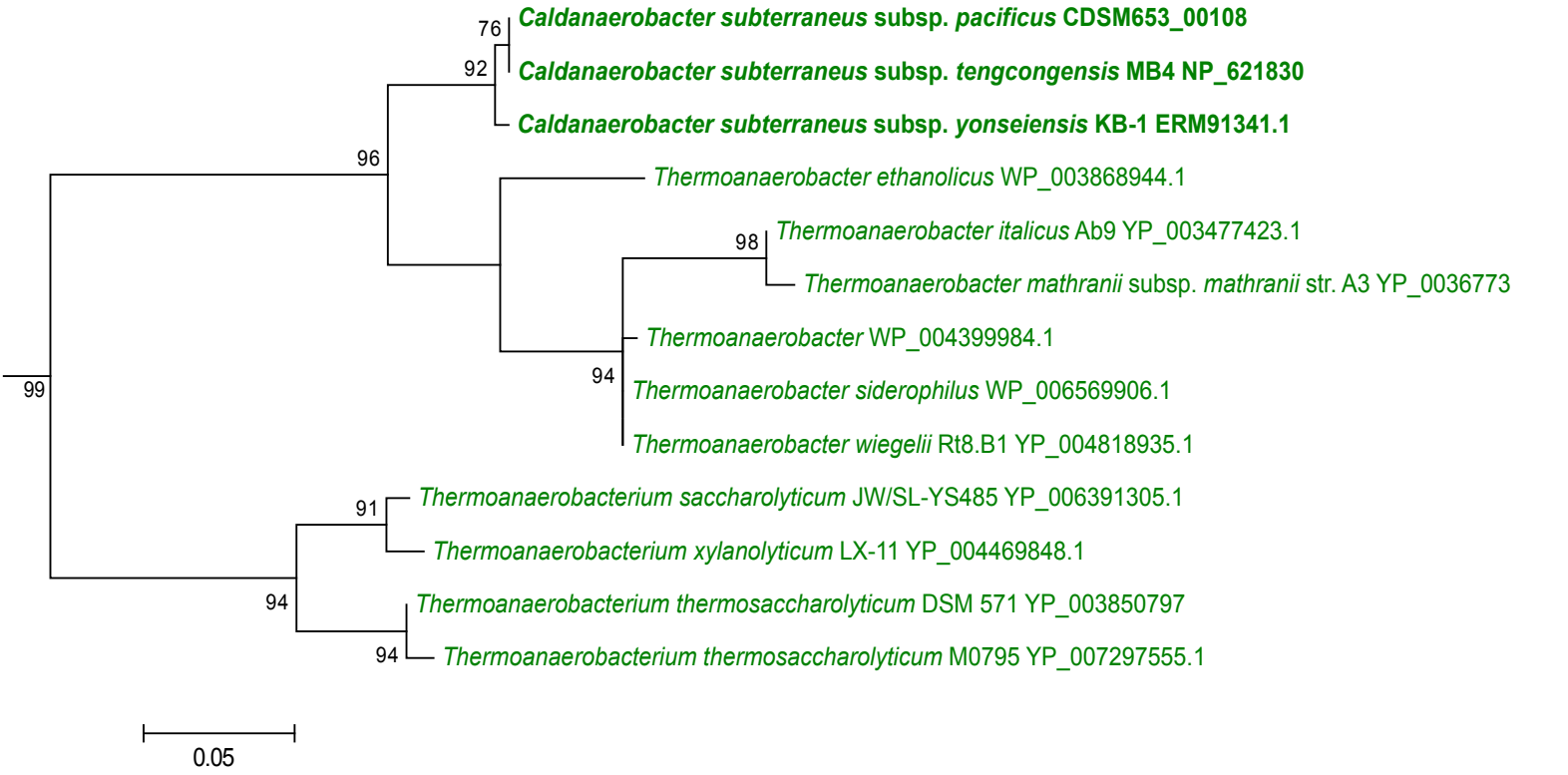

HypE

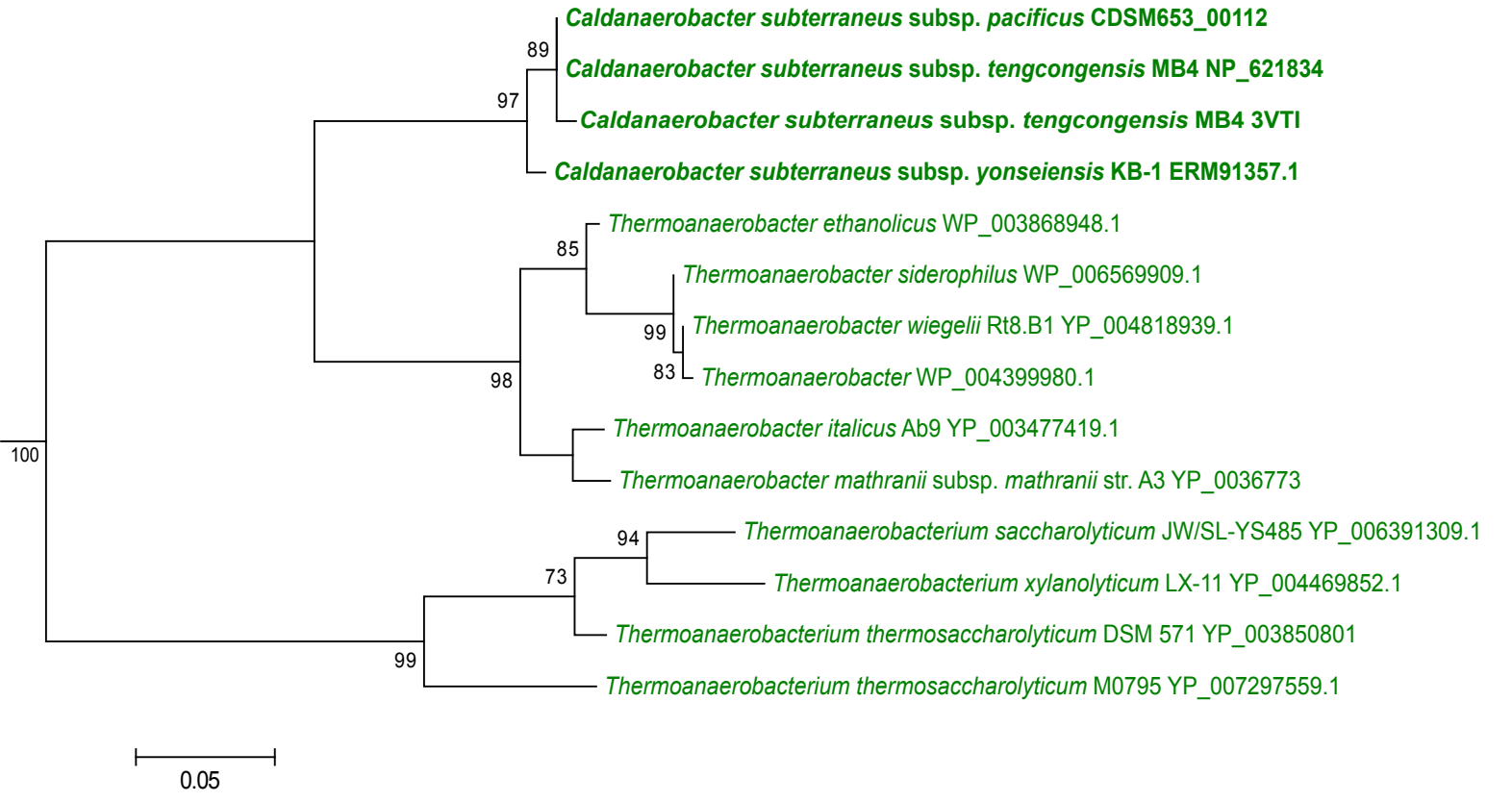

HypC

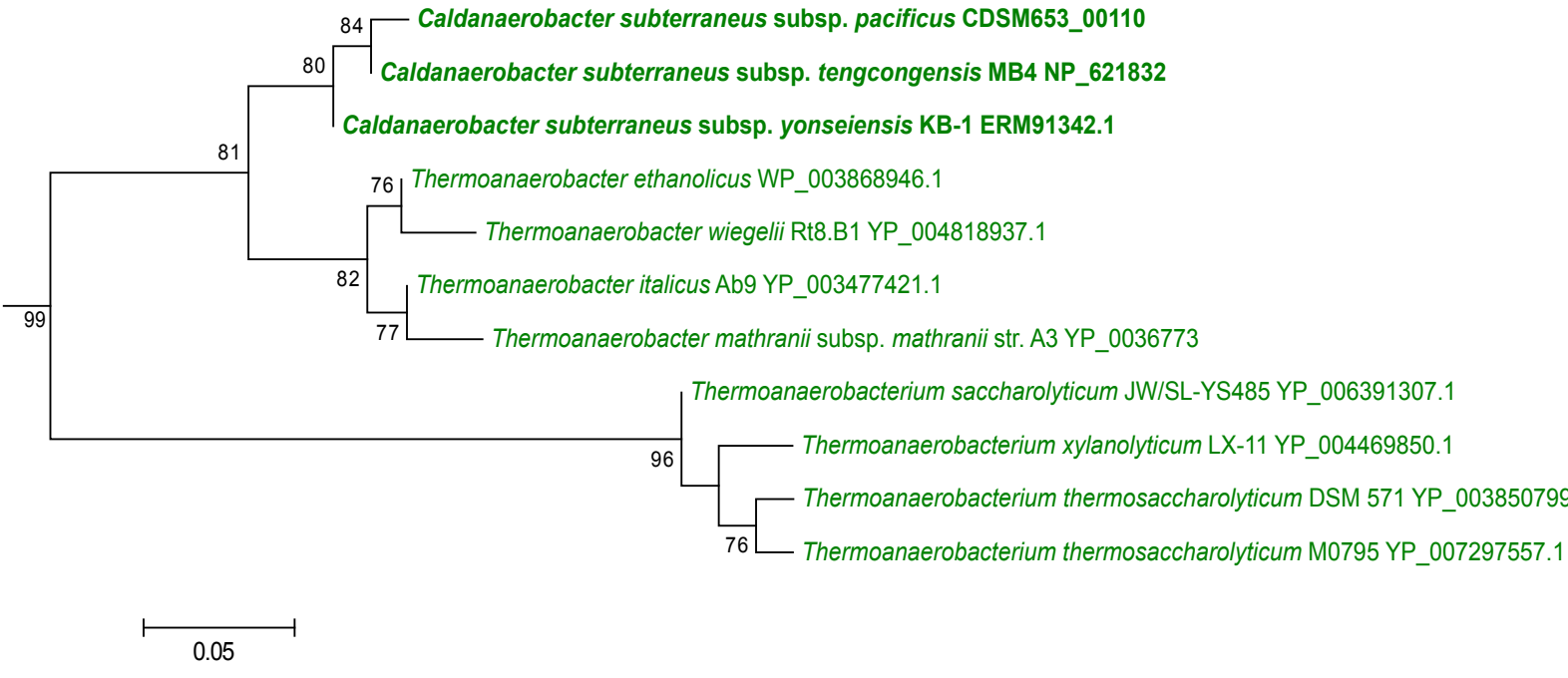

HypF

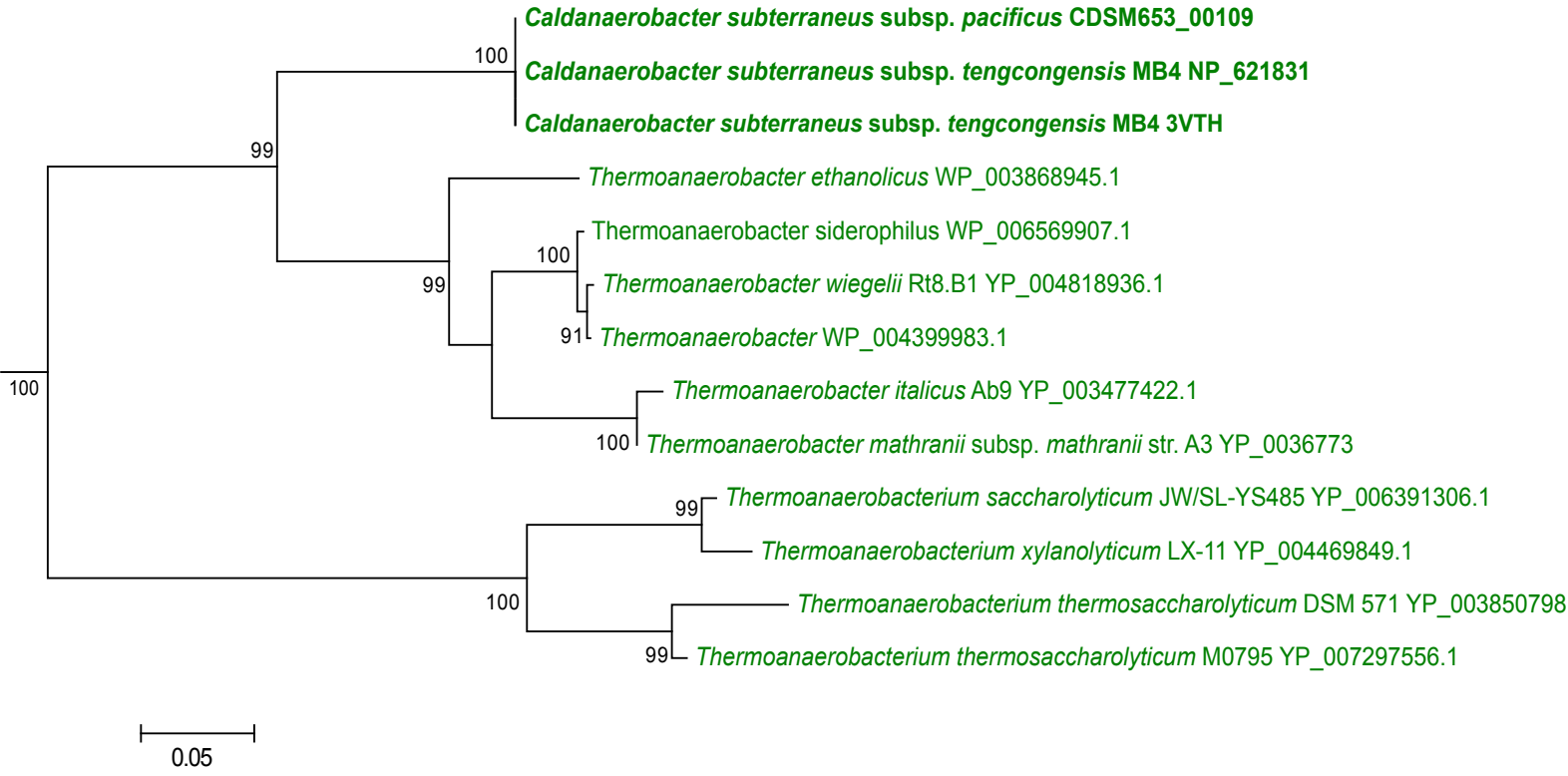

Supplement: Additional file 6: Figure S5. — Evolutive history of Hyp proteins from C. subterraneus subspecies. The subtrees were extracted from trees constructed using the maximum-likelihood method. Details are as shown in Additional file 3: Figure S2, unless specified otherwise. (PDF 58 kb) [file 12864_2015_1955_MOESM6_ESM.pdf]

HydA

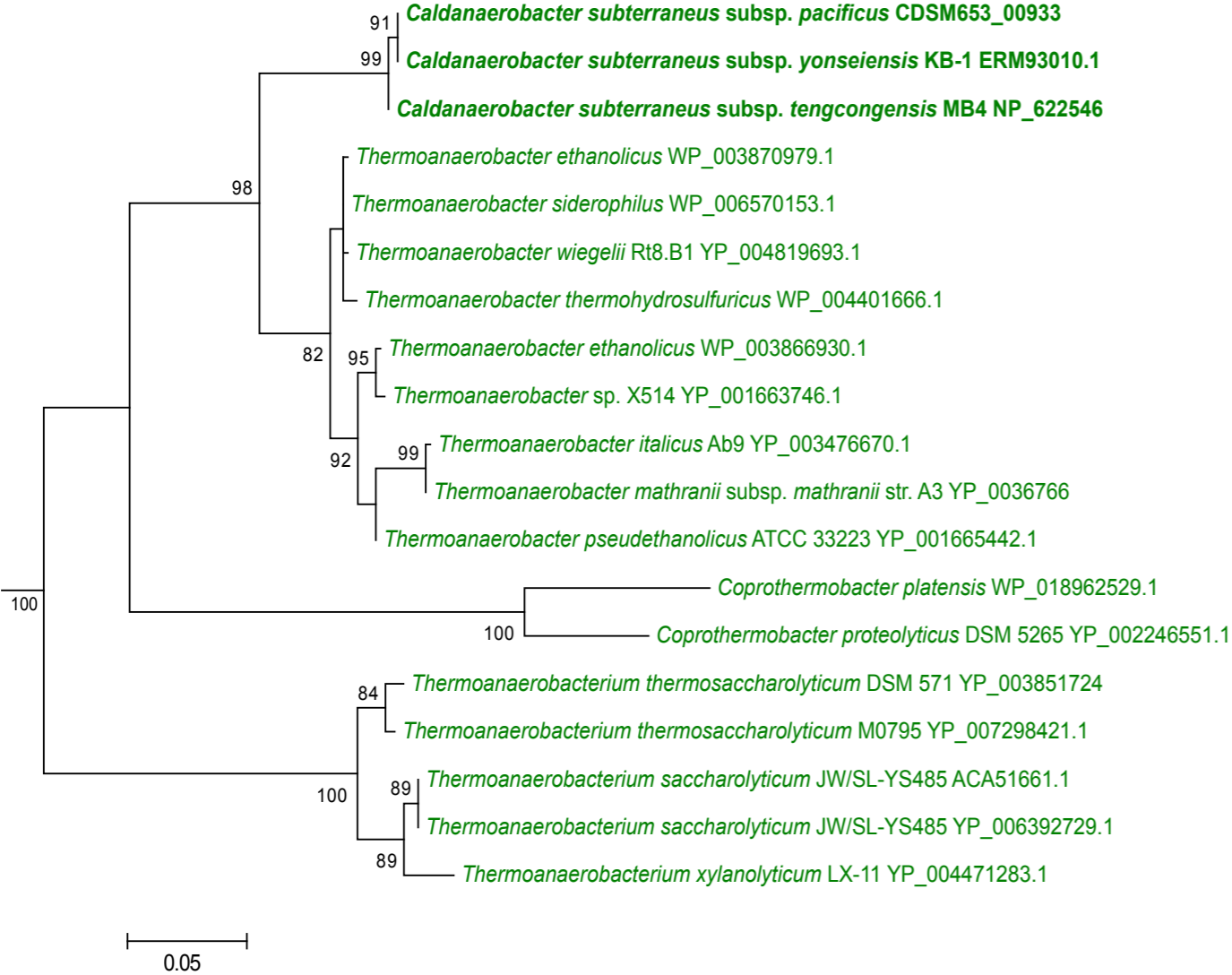

HydD

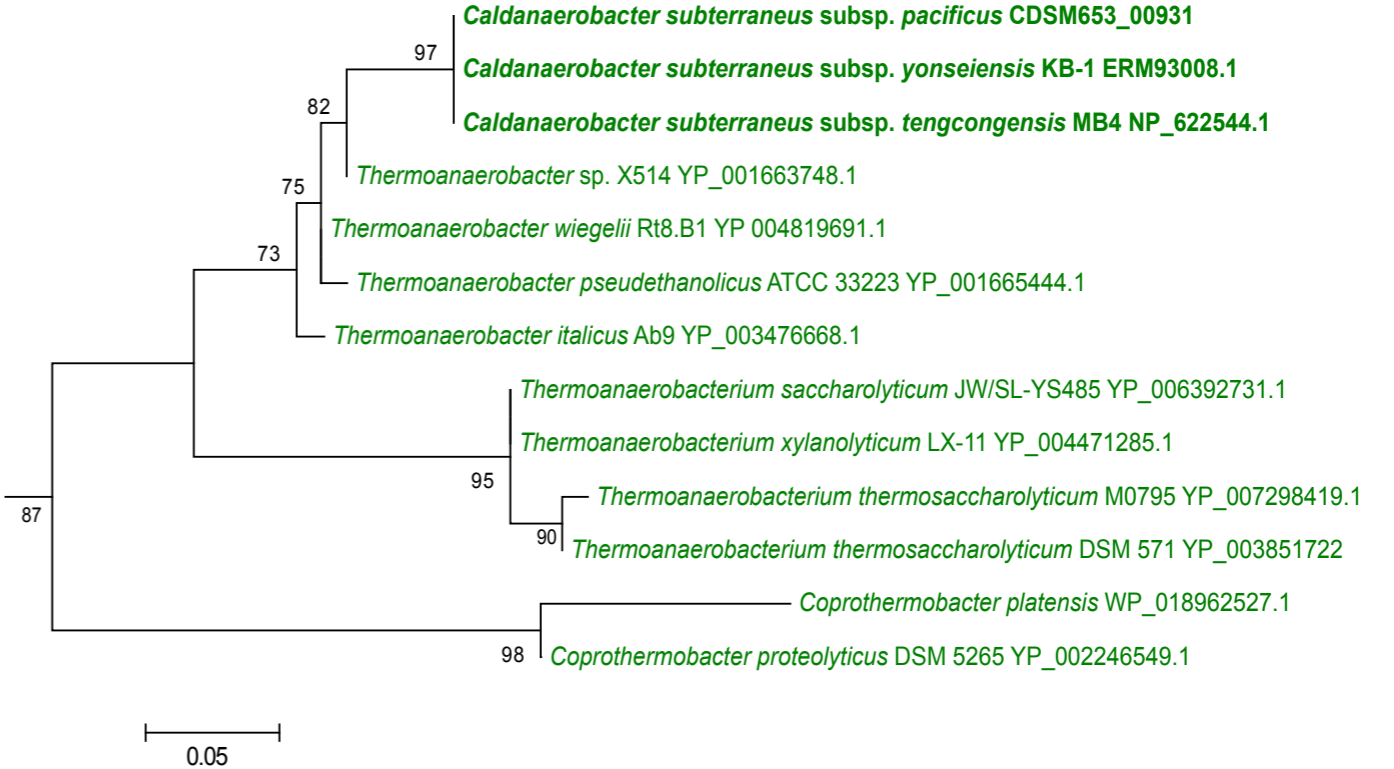

HydB

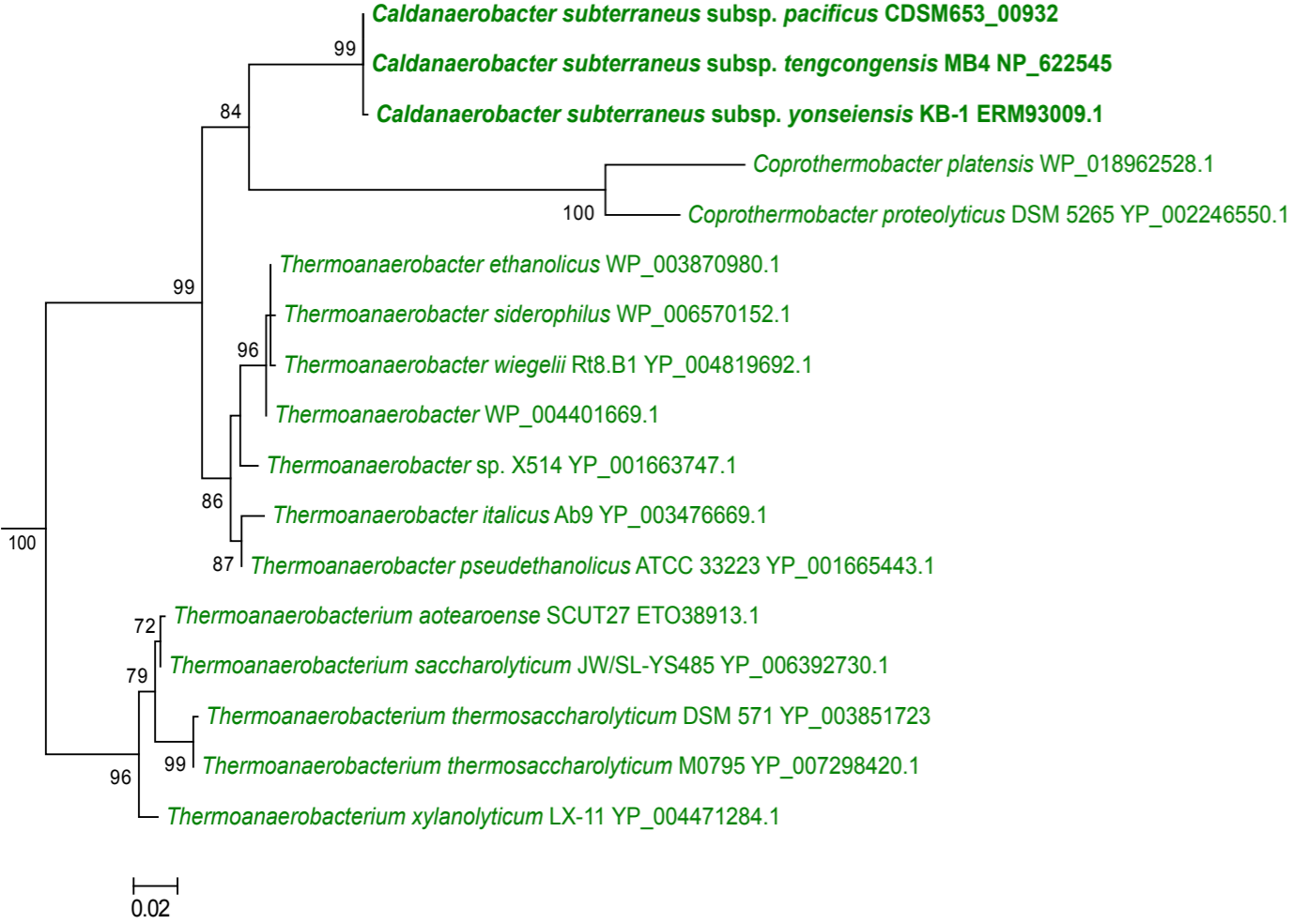

TTE0891

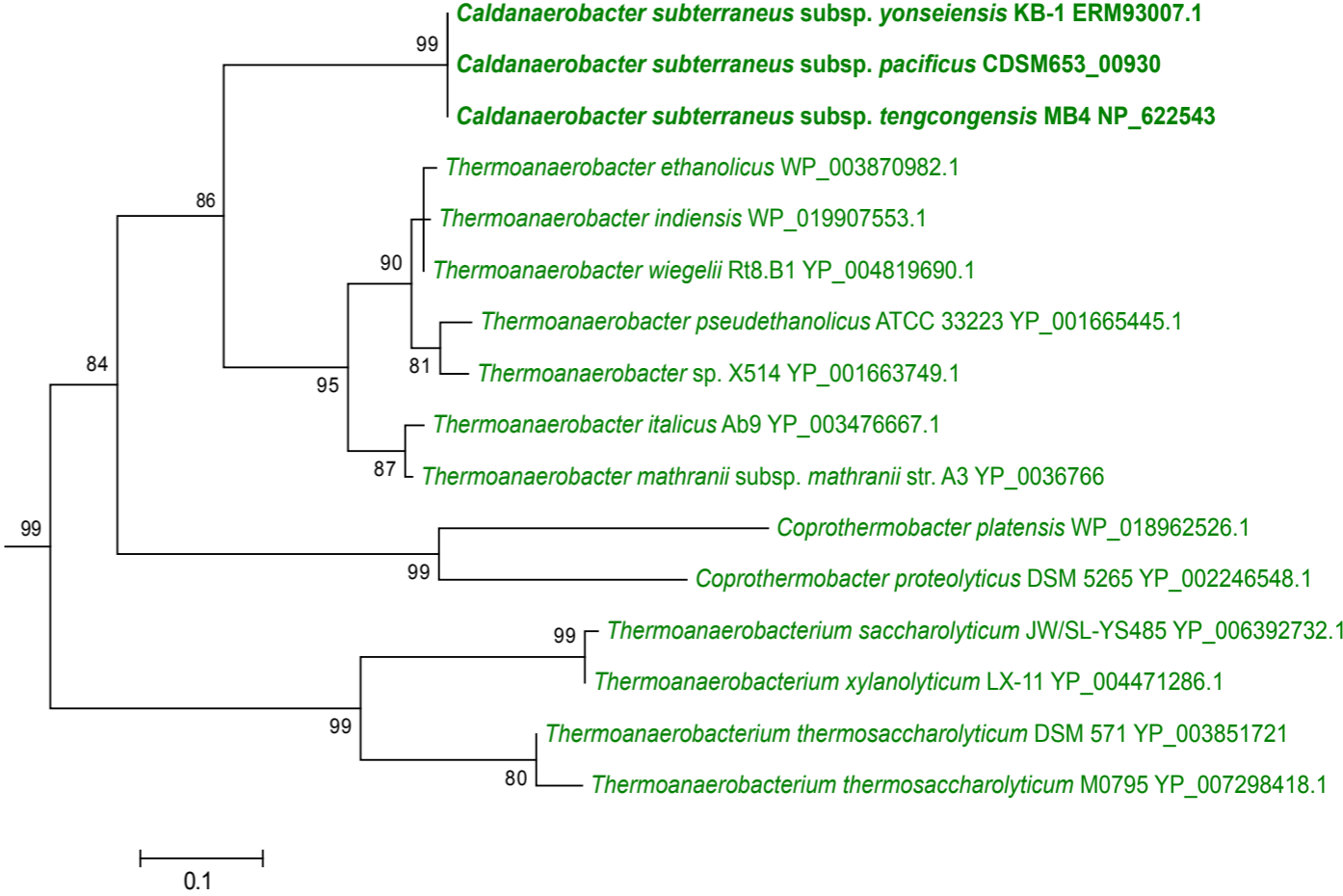

HydC

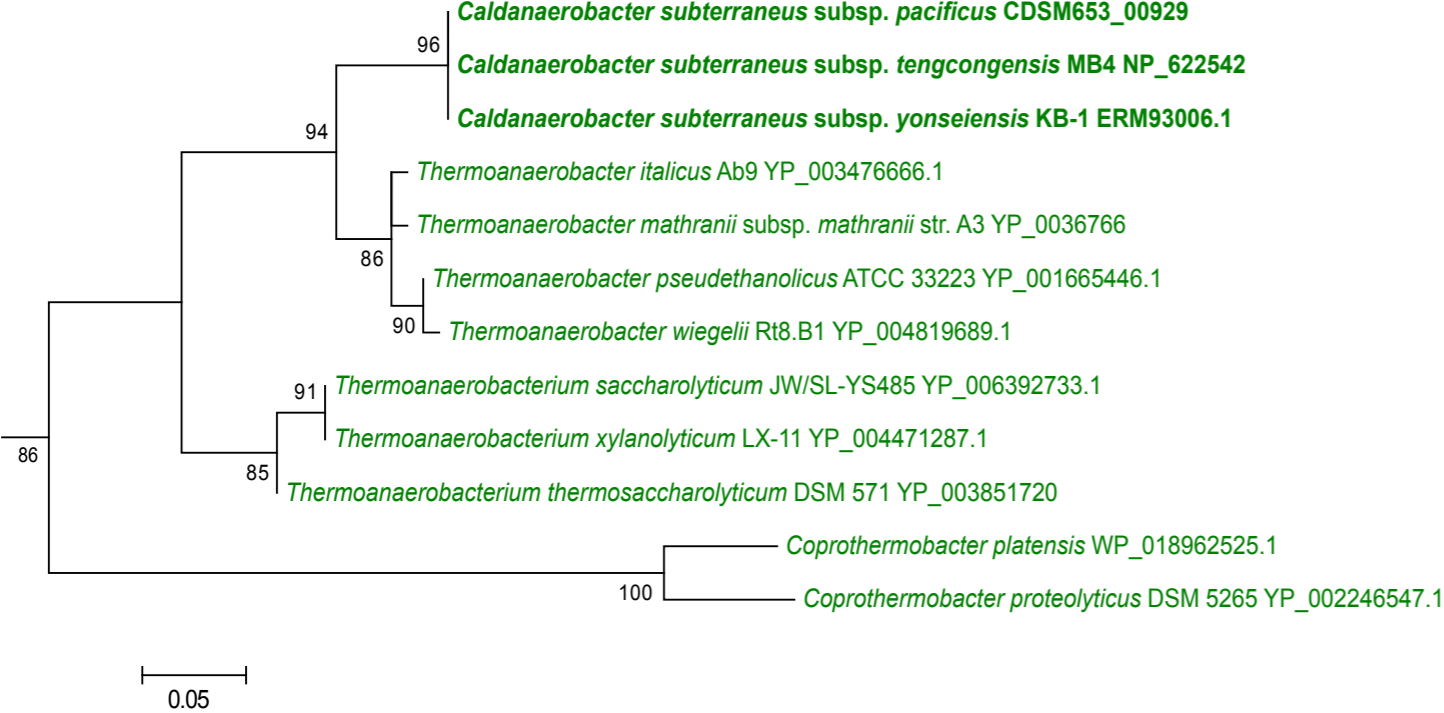

Supplement: Additional file 7: Figure S6. — Evolutive history of Hyd proteins from C. subterraneus subspecies. The subtrees were extracted from trees constructed using the maximum-likelihood method. Details are as shown in Additional file 3: Figure S2, unless specified otherwise. (PDF 56 kb) [file 12864_2015_1955_MOESM7_ESM.pdf]

MbxA

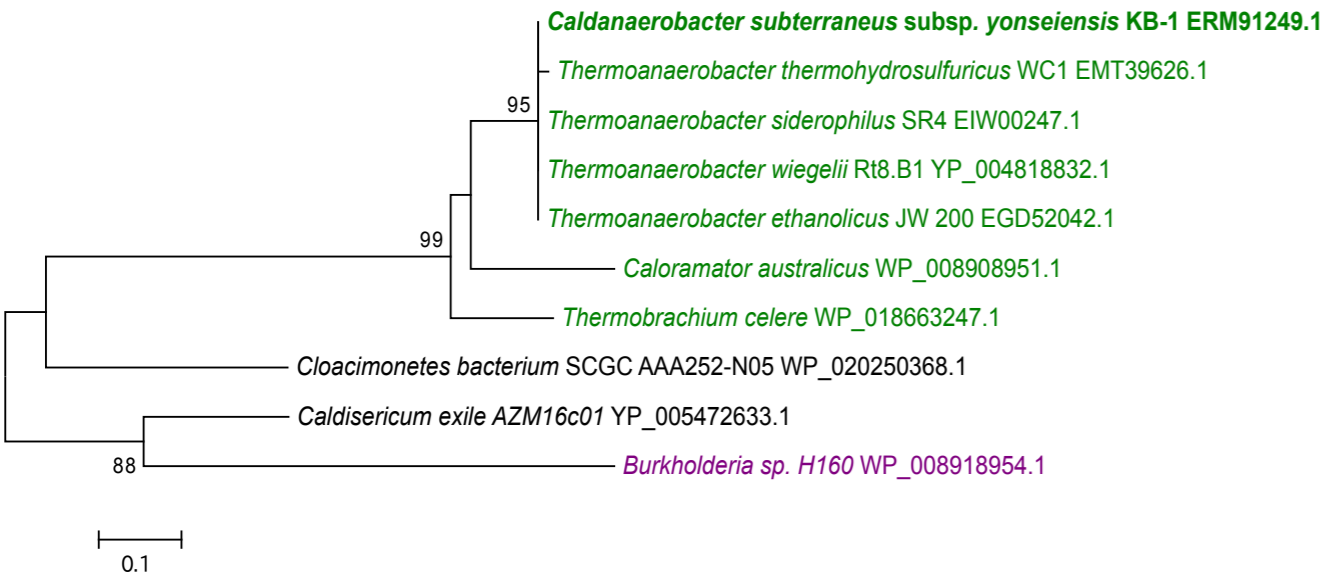

MbxB

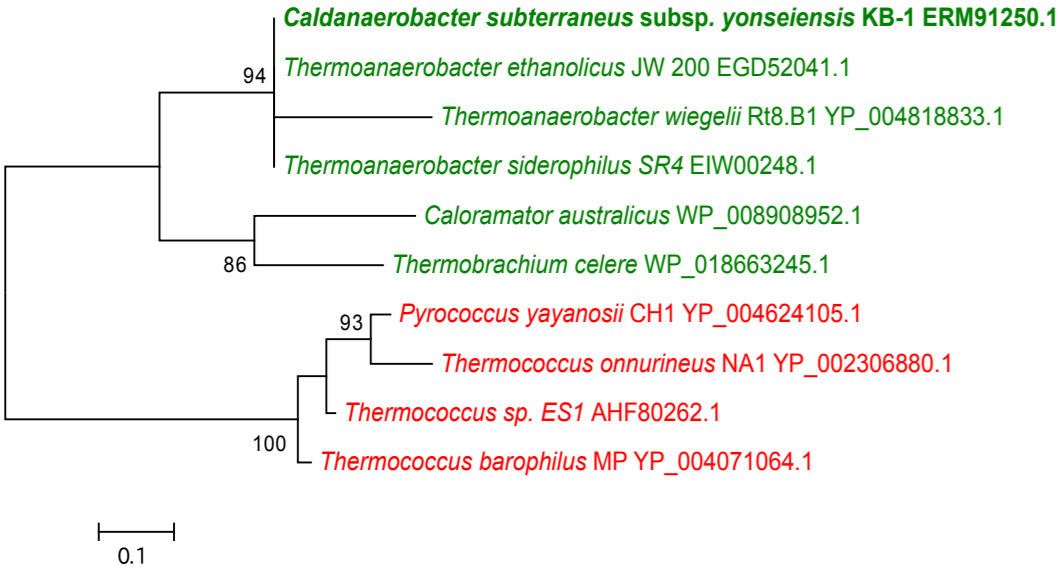

MbxC

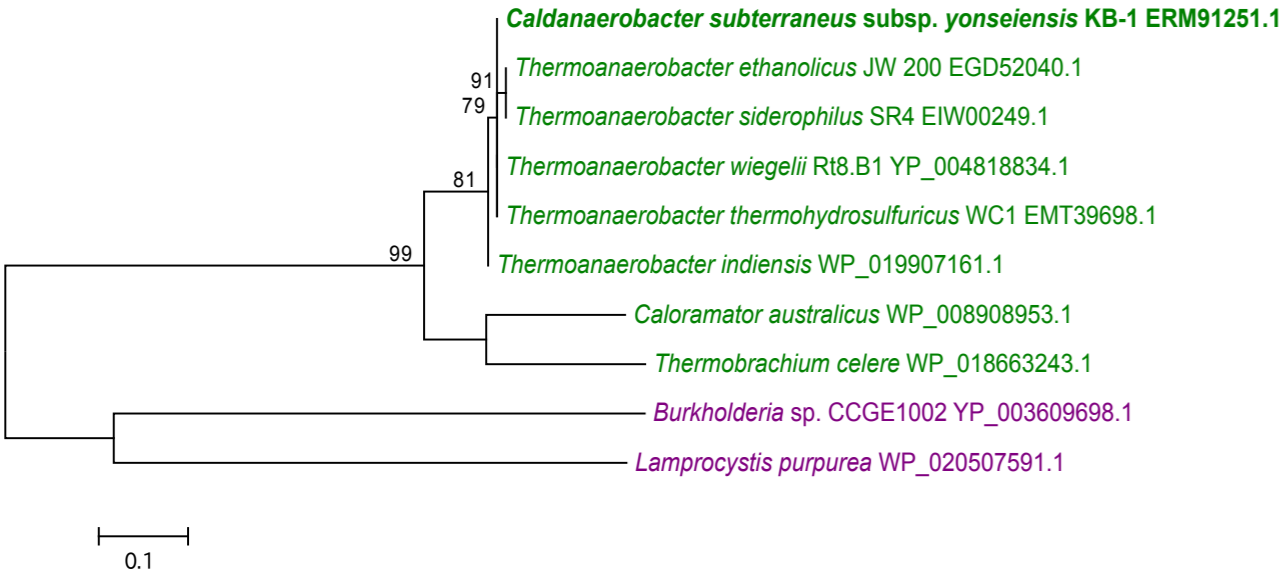

MbxD

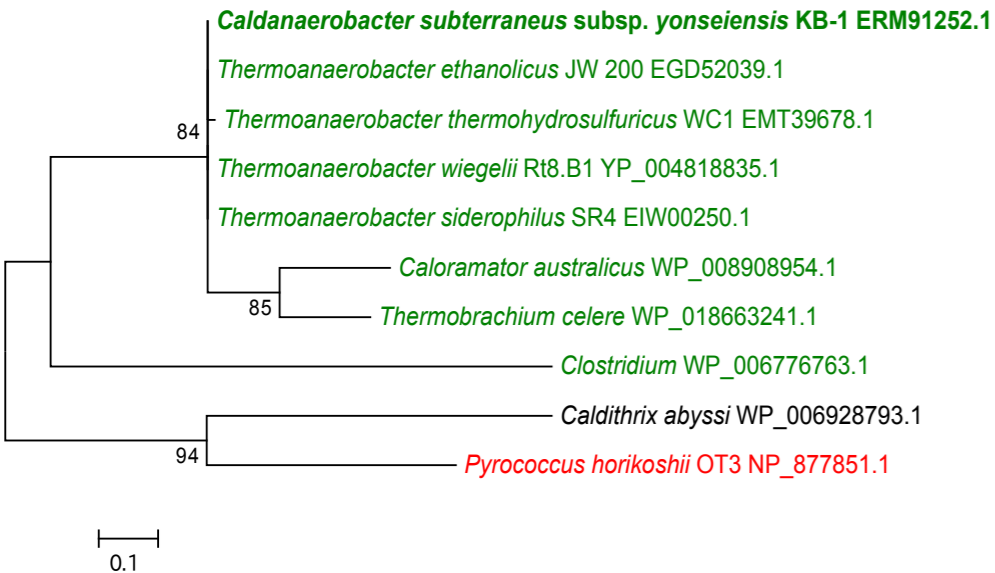

MbxF

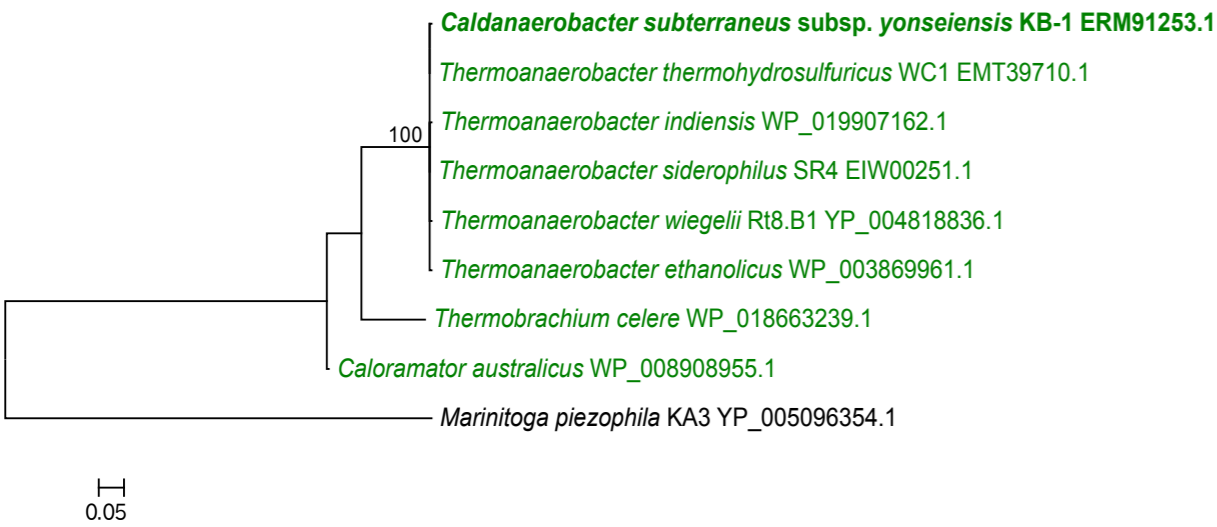

MbxG

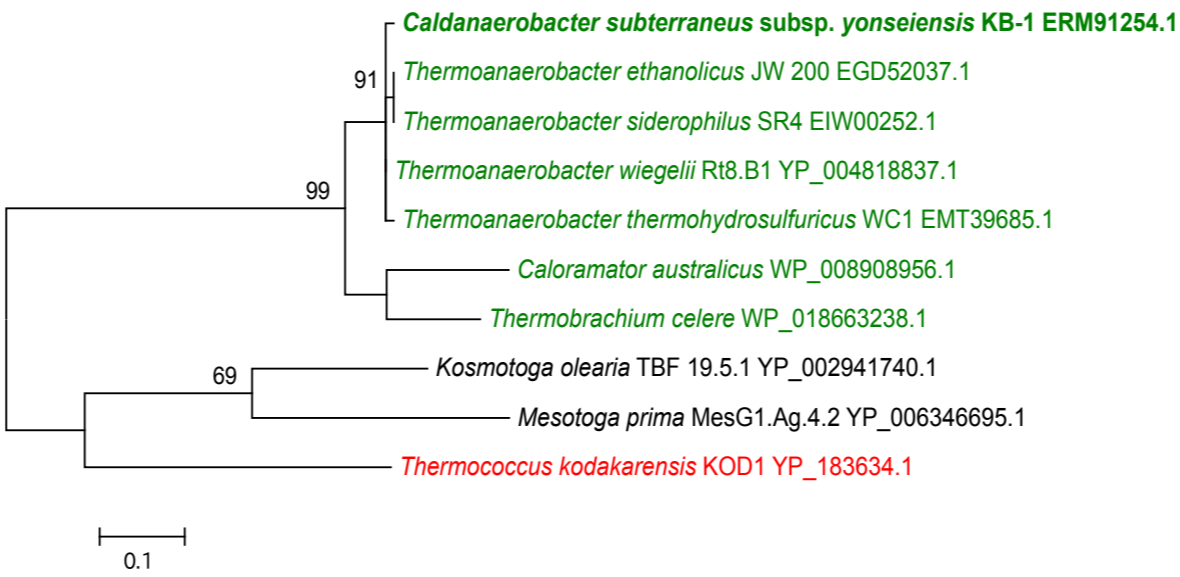

MbxH

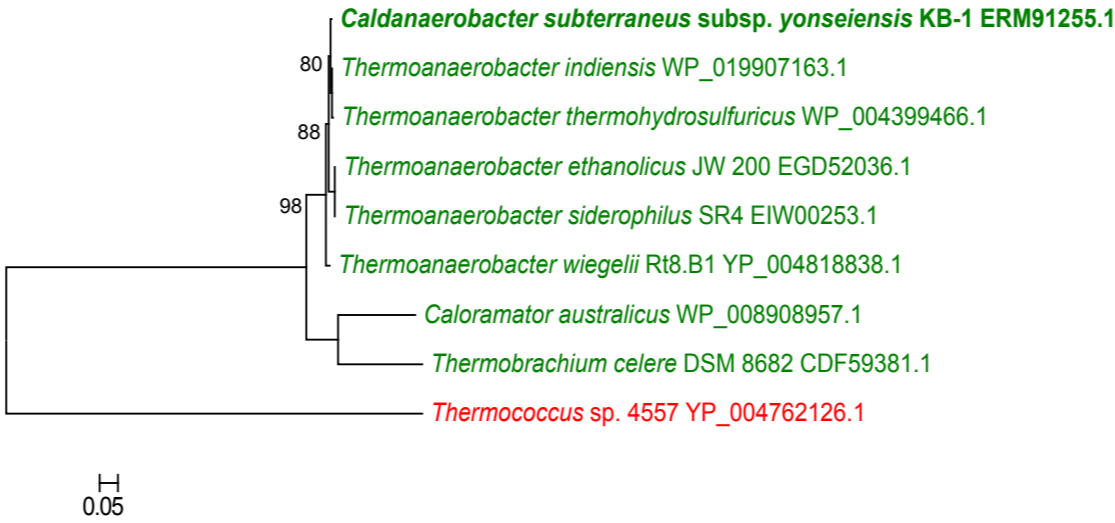

MbxH'

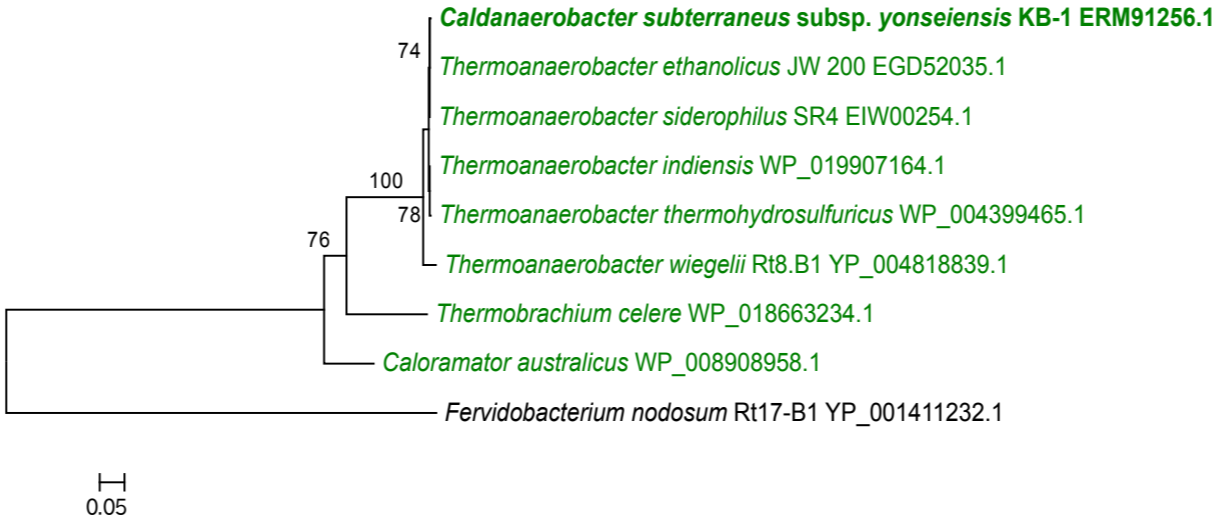

MbxJ

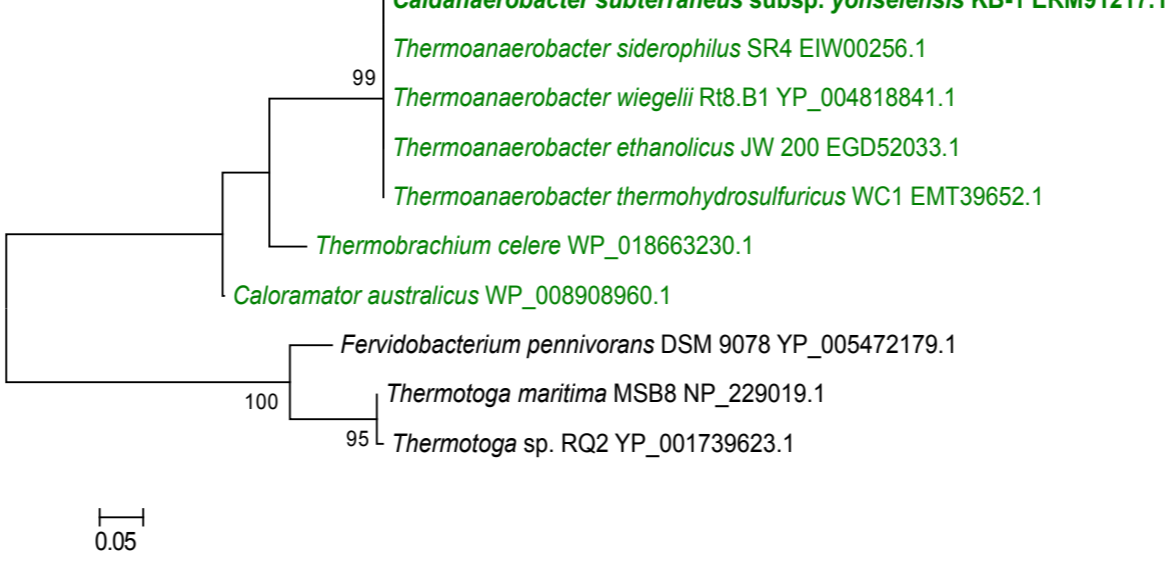

MbxK

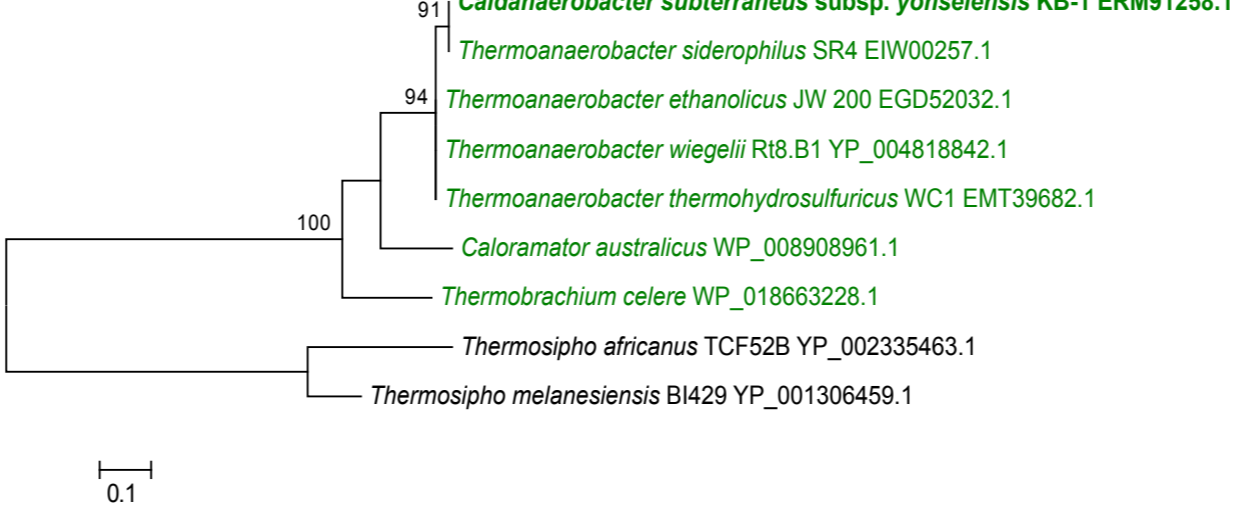

MbxL

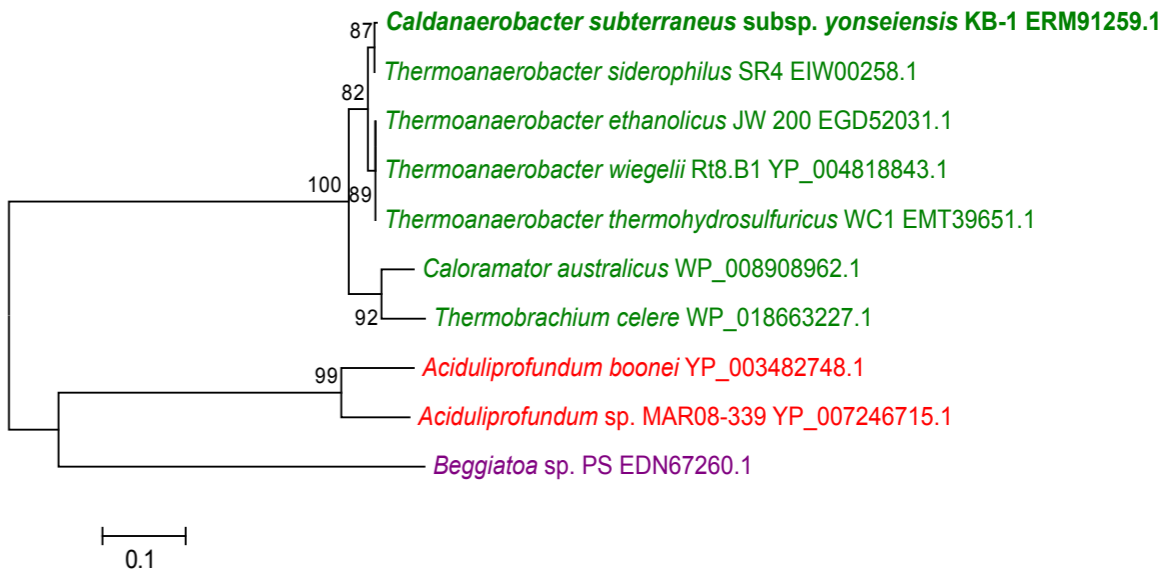

MbxM

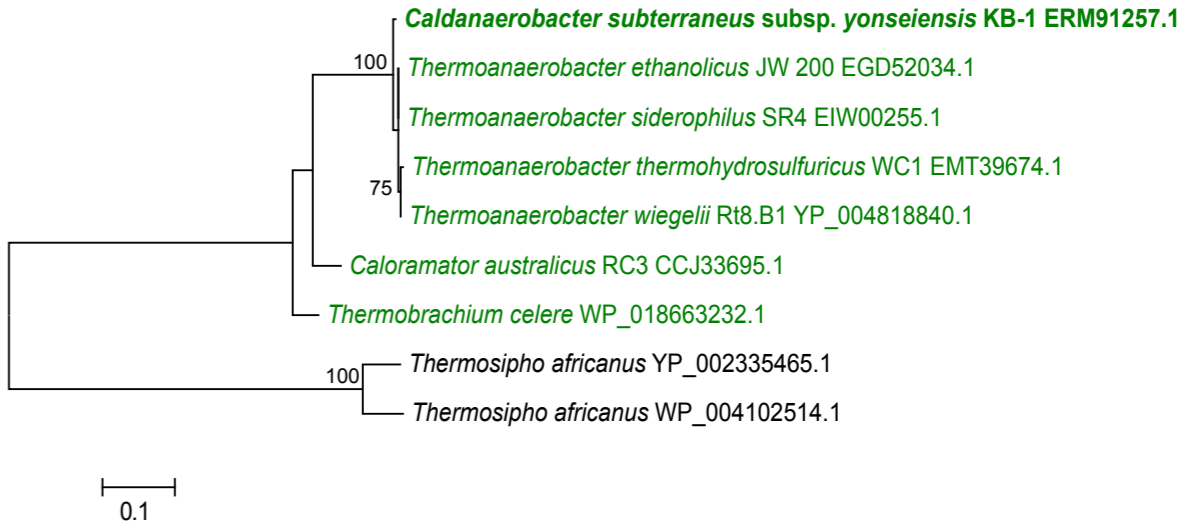

MbxN

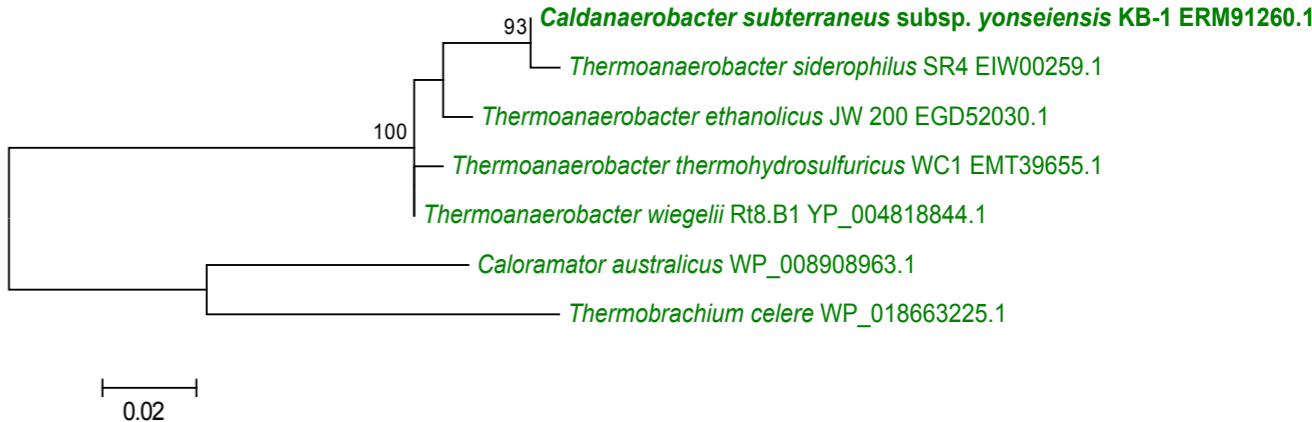

O163\_11495

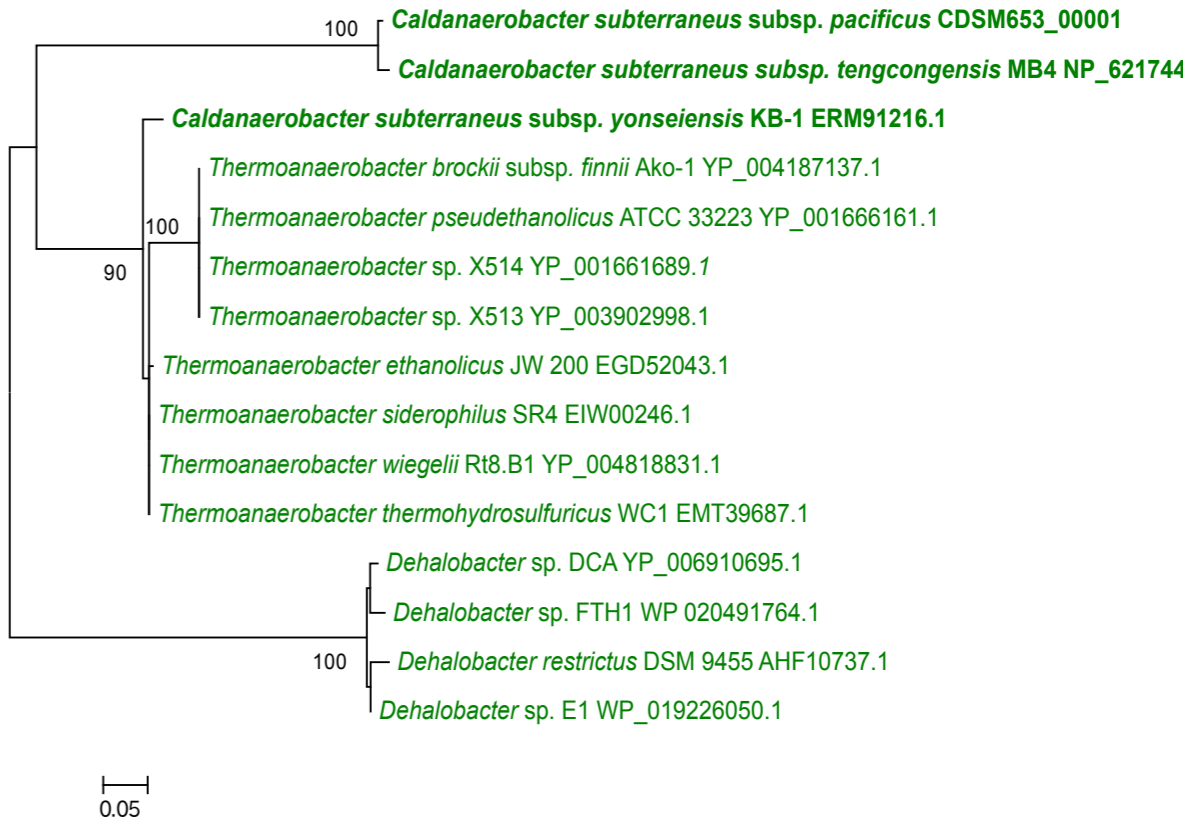

O163\_11565

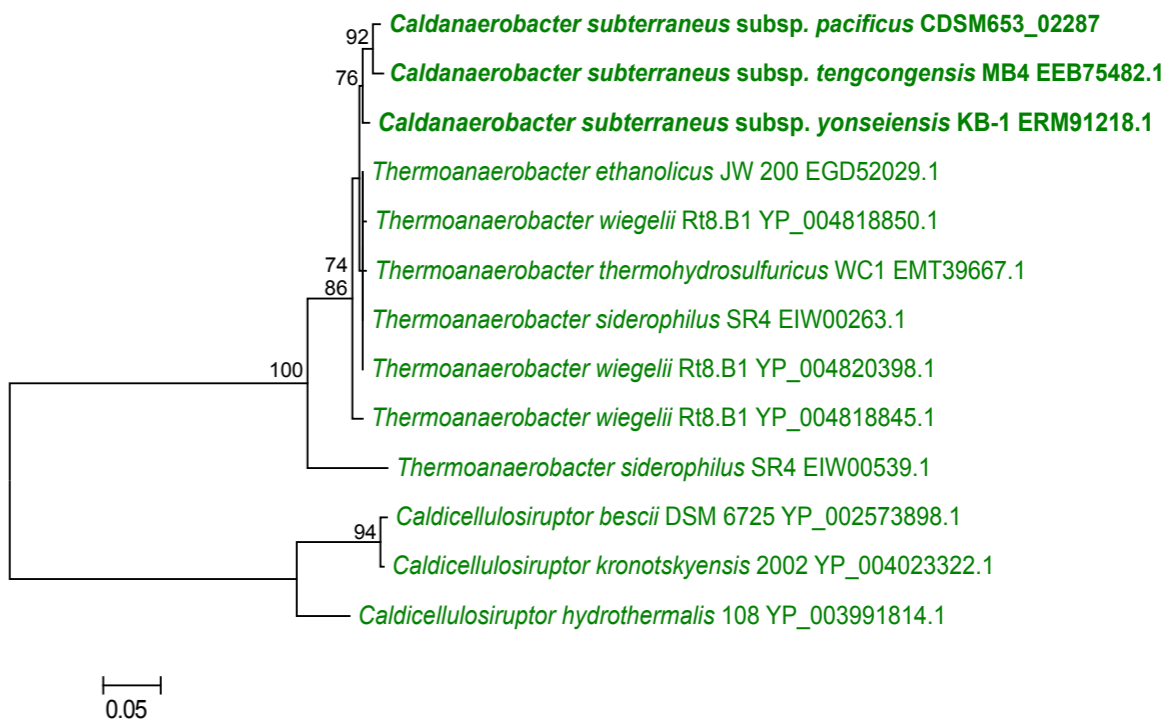

Supplement: Additional file 9: Figure S7. — Evolutive history of Mbx from C. subterraneus subsp. yonseiensis. Details are as shown in Additional file 3: Figure S2, unless specified otherwise. (PDF 70 kb) [file 12864_2015_1955_MOESM9_ESM.pdf]
